# Investigating CD8 T Cell Exhaustion in Chronic LCMV Infection by Flow Cytometry

**DOI:** 10.1002/cpz1.70445

**Published:** 2026-09-29

**Authors:** Dominique Stark, Marie‐Elen Tuchel, Gautier Stehli, Alessandro Genovese, Isaak Quast, Annette Oxenius

**Affiliations:** ^1^ Institute of Microbiology ETH Zurich Zurich Switzerland; ^2^ Department of Immunology Monash University Melbourne Victoria Australia

**Keywords:** adoptive cell transfer, CD8 T cell exhaustion, chronic viral infection, high‐parameter flow cytometry, LCMV

## Abstract

CD8 T cells and the process of T cell exhaustion are central topics in both basic immunology and translational research, including clinical applications. Here, we provide a comprehensive, step‐by‐step guide to investigate CD8 T cells in murine *in vivo* lymphocytic choriomeningitis virus (LCMV) infection models. We outline viral stock preparation, infection, and viral titer evaluation. We put an emphasis on flow cytometry as a method to investigate phenotypical and functional characteristics of CD8 T cell exhaustion, but the protocols provided will also facilitate imaging or transcriptomic analyses. With these resources, we aim to streamline establishment of the LCMV model for new users and to support harmonization and optimization of workflows for laboratories already working with this model. © 2026 The Author(s). *Current Protocols* published by Wiley Periodicals LLC.

**Basic Protocol 1**: Virus production

**Basic Protocol 2**: Virus titer quantification from virus stocks by focus‐forming assay

**Alternate Protocol 1**: Virus titer quantification from infected tissues by focus‐forming assay

**Basic Protocol 3**: Peptide‐MHC‐I tetramer production

**Basic Protocol 4**: Naïve CD8 T cell isolation using StemCell^TM^ kit

**Alternate Protocol 2**: Naïve CD8 T cell enrichment using MojoSort^TM^ kit

**Basic Protocol 5**: Adoptive CD8 T cell transfer and LCMV infection

**Basic Protocol 6**: Animal husbandry

**Basic Protocol 7**: Preparation of single‐cell suspension from secondary lymphoid organs

**Alternate Protocol 3**: Preparation of single‐cell suspension from peripheral tissues

**Basic Protocol 8**: Extracellular and intracellular staining

**Basic Protocol 9**: Restimulation analysis

**Basic Protocol 10**: Sorting

## INTRODUCTION

Clearing pathogens, preventing infectious diseases, and eliminating mutated cells that may give rise to tumors are central functions of the immune system. An effective immune response can control and eliminate pathogens, such as in influenza virus in acute infection, within days (Miao et al., 2010; Smith et al., 2018). In contrast, persistent infections and life‐threatening cancers result from a heavily regulated and attenuated immune response (McLane et al., 2019).

CD8 T cells play a central role in mammalian adaptive immunity by recognizing and killing infected or tumorigenic cells. In persistent infections and cancers, the ongoing presence of foreign antigens or neo‐antigens, respectively, leads to constant activation of antigen‐specific CD8 T cells, resulting in the attenuation of their effector functions (Shin & Wherry, 2007). This phenomenon, first described in 1993 by Moskophidis et al. (1993), is called “T cell exhaustion” to account for the gradual loss of effector functions, including the secretion of IL‐2 and IFN‐γ and direct killing via perforin and granzyme (Baessler & Vignali, 2024; McLane et al., 2019; Shin & Wherry, 2007; Wherry & Kurachi, 2015). Additionally, antigen‐specific CD8 T cells facing persistent antigen encounter lose epigenetic plasticity (Abdel‐Hakeem et al., 2021; Belk et al., 2022; Yates et al., 2021), acquire DNA damage (Koufaris et al., 2025; Rivadeneira et al., 2025), and have altered metabolism (Bengsch et al., 2016; Richter et al., 2024; Salazar‐Roa & Malumbres, 2017; Vardhana et al., 2020), all of which contribute to the semblance of a “fatigued” cell that runs out of “fuel.”

Importantly, more recent research has shown that T cell exhaustion represents an actively regulated differentiation program rather than a mere loss of function and that limiting immunopathology is likely a central reason for this adaptation (Cornberg et al., 2013; Frebel et al., 2012).

Therefore, it is of fundamental interest to understand the mechanisms that lead to the adaptation of CD8 T cells to persistent antigen exposure. Such insights hold strong translational relevance, as almost all immune cell–based cancer treatments, including chimeric antigen receptor (CAR) T cell therapy and immune checkpoint blockade, rely on a functional pool of cytotoxic CD8 T cells (Codarri Deak et al., 2022; Gumber & Wang, 2022; Tichet et al., 2023; Yu et al., 2026). To empirically investigate the mechanisms of T cell exhaustion, a robust experimental model is required. Here, we describe murine lymphocytic choriomeningitis virus (LCMV) infection, a widely used and well‐established model system to study both acute and persistent viral infections (Abdel‐Hakeem, 2019; Oldstone, 2013). The article provides a step‐by‐step guide from virus production to host infection and experimental readouts (Fig. 1).

**Figure 1:**
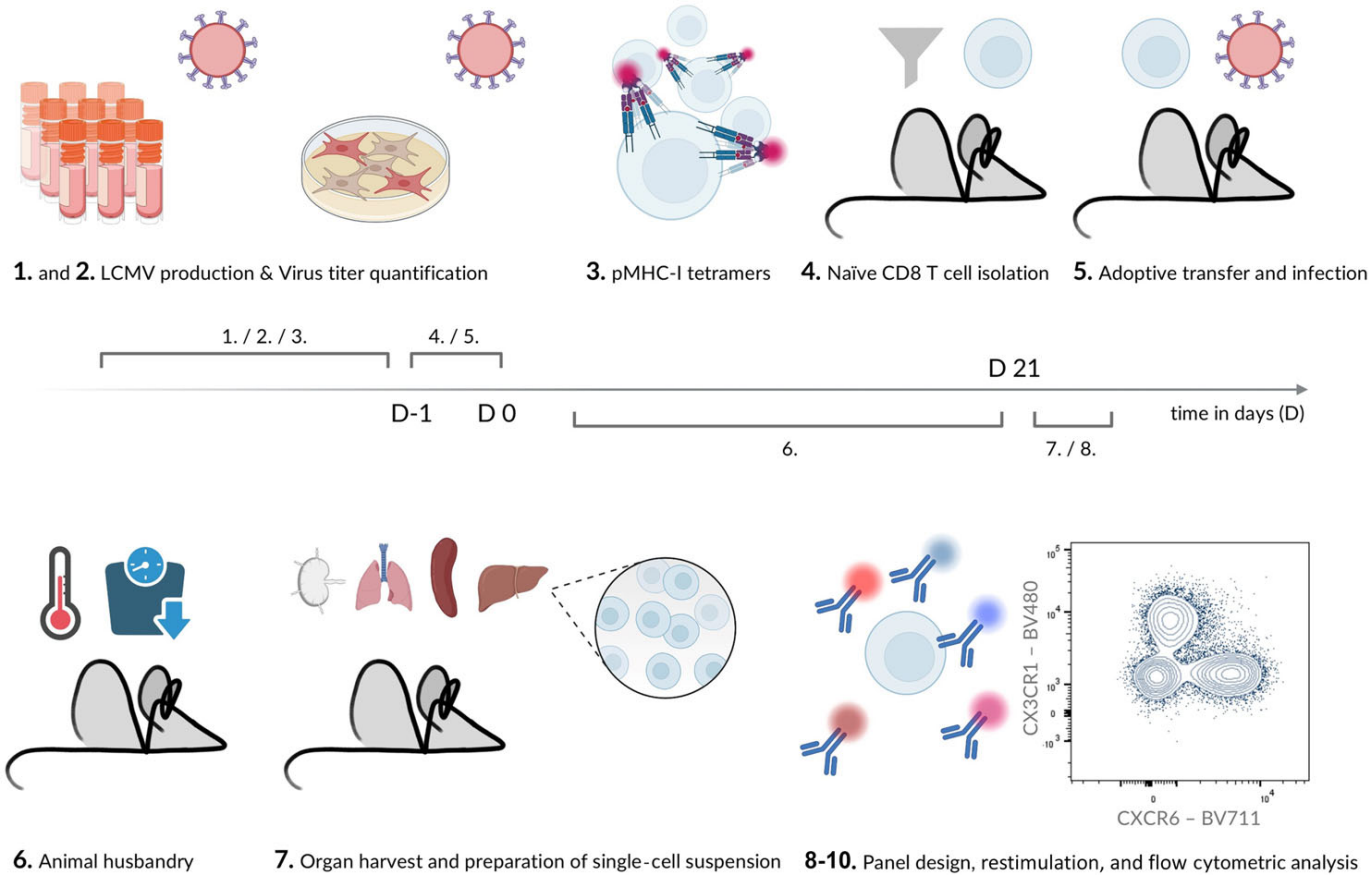
Graphical summary of the provided protocols. The whole workflow of establishing a chronic LCMV infection in mice is split into eight parts that correspond to the basic protocols. Basic Protocols 1 to 4 apply to procedures before the infection, followed by optional adoptive cell transfer and the infection itself in Basic Protocol 5. Basic Protocols 7 to 10 contain guidelines for post‐experimental data analysis. Animal monitoring during the infection is discussed in Basic Protocol 6. Graphics were generated with BioRender.

The focus is primarily on CD8 T cells in the context of chronic viral infections, as this is the cell type in which T cell exhaustion is best characterized. It is worth noting that similar concepts have been shown for CD4 T cells and that B cell responses contribute to LCMV control (Crawford et al., 2014; Planz et al., 1997; Stoycheva et al., 2021; Xia et al., 2022).

Finally, the concept of adoptive cell transfer of LCMV‐specific CD8 T cells is discussed, as this enables the introduction of genetic modifications exclusively in the cells of interest without genetically altering the host.

### Lymphocytic Choriomeningitis Virus (LCMV)

LCMV is a non‐cytopathic, small, single‐stranded RNA virus from the arenavirus family. It contains two RNA strands, named S and L segments. The S segment encodes for the two main structural proteins: the glycoprotein complex (GPC), which is exposed on the viral envelope and is responsible for host cell attachment and fusion, and the nucleoprotein (NP), which is internal and encapsulates the RNA. The L segment encodes a RING‐finger protein (Z polypeptide), which is essential for virus assembly and budding, and a polymerase, which is critical for viral replication (Buchmeier et al., 1980; Moskophidis et al., 1993; Rojek et al., 2008) (Fig. 2).

**Figure 2:**
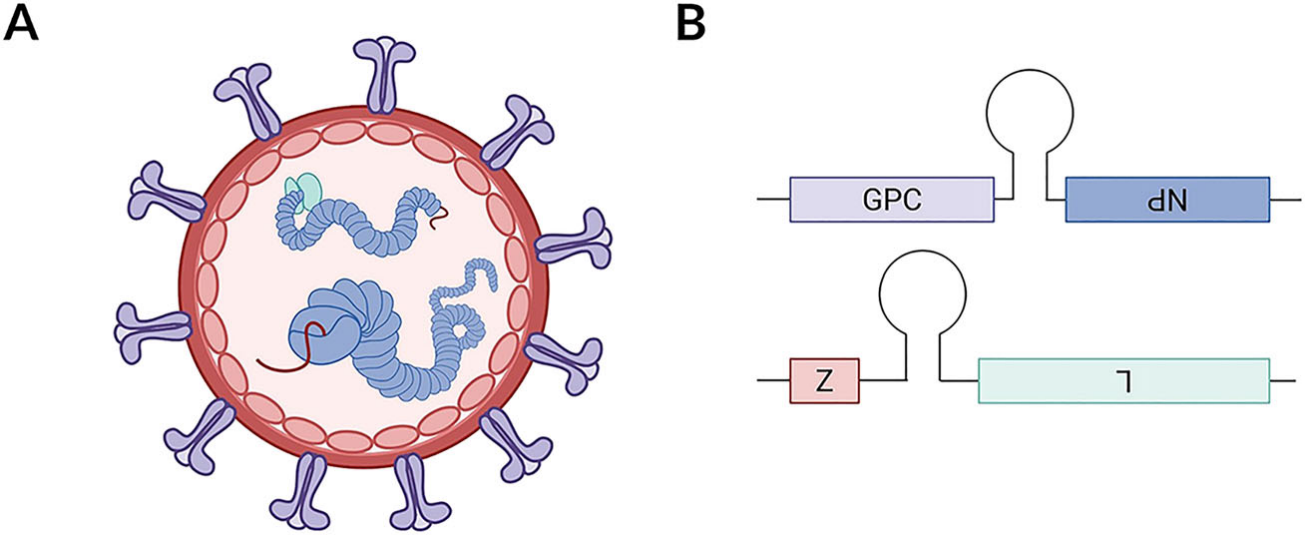
Lymphocytic choriomeningitis virus (LCMV). (**A**) Schematic representation of an LCMV virion with its two major proteins: the glycoprotein complex (GPC, in purple) and the nucleoprotein (NP, in blue). (**B**) Illustration of LCMV's genome, with the four coding regions for the GPC, NP, the L polymerase, and the Z polypeptide. Graphics were generated with BioRender.

### LCMV Tropism

Most viral infections are controlled quickly due to rapidly reacting innate and adaptive immune responses (Miao et al., 2010; Wherry et al., 2003). Virus antigen–specific CD8 T cells can proliferate extremely fast upon T cell receptor (TCR)‐mediated recognition of the cognate peptide antigen presented on major histocompatibility complex I (MHC‐I). This results in the generation of a large number of cytotoxic CD8 T cells within days after infection to exert pressure on the pathogen. Some viruses (e.g., cytomegalovirus) have developed strategies to evade the CD8 T cell response by downregulating host MHC‐I molecules in infected cells, which undermines antigen presentation and recognition (Halenius et al., 2015; Zangger & Oxenius, 2022). Such immune evasion strategies, including viral latency, can lead to persistent viral infections but do not necessarily result in T cell exhaustion. Antigen exposure in these situations is sporadic and less pronounced than during active chronic viral infections that do not evade CD8 T cell immunity (Baessler & Vignali, 2024; Pan et al., 2026; Vardhana et al., 2020). Active chronic viral infections like LCMV infection in mice lead to an adaptation of the CD8 T cell response by two means. First, the virus infects a wide range of cell types in most tissues, including the kidneys, lungs, liver, heart, thymus, secondary lymphoid organs, blood endothelium, and, as the virus's name indicates, central nervous system (Ahmed & Oldstone, 1988; Wherry et al., 2003). Thus, LCMV constitutes a systemic infection, with its broad tropism enabled by the use of widely expressed receptors for host cell entry (e.g., α‑dystroglycan or heparan sulfate proteoglycans) (Kunz et al., 2001; Smelt et al., 2001; Volland et al., 2021). Second, LCMV replication is extremely efficient, resulting in rapid transmission to new host cells. In comparison to other persistent viral infections, such as infection with cytomegalovirus (CMV), LCMV does not actively evade the host CD8 T cell response but rather outpaces the response by spreading faster than infected cells get cleared. Viral persistence leads to continuous antigen presentation and TCR triggering, which drives T cell exhaustion.

There are several strains of LCMV that share almost identical genetic information, with only a few nucleotide alterations. These differences are localized to a) the α‐dystroglycan‐binding domain of the GPC and b) the polymerase, with both changing the fitness of the virus (Sullivan et al., 2011). As a consequence of altered cell tropism and replication speed, certain LCMV strains do not establish a persistent infection but rather are rapidly controlled, making LCMV an ideal model system to study and compare T cell responses in acute and chronic viral infections (Althaus et al., 2007; Norris et al., 2013; Yoo et al., 2023).

### Murine LCMV Infection Model to Study Acute and Chronic Viral Infections

There are numerous different LCMV strains, but the four strains that are frequently used (Table 1) to study acute and chronic infections are LCMV Armstrong (Arm) and its derivate LCMV Clone 13 (CL13) as well as LCMV WE and its derivative LCMV Docile (Doc). LCMV Arm and WE elicit an acute infection regardless of the dose of the inoculum. LCMV CL13 and Doc also get controlled when mice are infected with a low‐dose inoculum (≤10^3^ focus‐forming units, or ffu) but reach viral persistence at a higher infection dose (≥10^6^ ffu) (Ahmed et al., 1984; Moskophidis et al., 1993). Focusing on LCMV Arm and CL13, the difference in viral persistence can be partially attributed to the lower binding affinity of LCMV Arm for α‐dystroglycan, which alters viral tropism and limits infection of dendritic cells. Efficient infection of dendritic cells by LCMV CL13 is thought to promote viral persistence by impairing dendritic cell function and antiviral immune priming. Furthermore, LCMV Arm lacks the gain‐of‐function polymerase mutation of LCMV CL13, resulting in reduced replicative capacity and less efficient viral propagation (Sevilla et al., 2003; Sullivan et al., 2011). Comparing LCMV WE and Doc, LCMV Doc propagates more slowly during the initial phase of the infection, resulting in reduced innate immune activation and less type I IFN production. The resulting delay in antiviral immune priming provides LCMV Doc with a critical head start, allowing the virus to establish persistence before effective immune control can be achieved (Xu et al., 2021).

**Table 1:** LCMV Strains Frequently Used for *In Vivo* Studies

Name	Alias	Parental strain	Dose for acute infection	Establishes persistence	Dose for chronic infection	Cell line for virus production
Armstrong	Arm	‐	2 × 10^5^ ffu	No	‐	BHK‐21
WE	WE	‐	2 × 10^3^ – 2 × 10^5^ ffu	No	‐	L‐929
Clone 13	CL13	Armstrong	2 × 10^2^ – 2 × 10^3^ ffu	Yes	2 × 10^6^ ffu	BHK‐21
Docile	Doc	WE	‐	Yes	2 × 10^6^ ffu	MDCK / BHK‐21

### CD8 T Cell Response to Viral Infections

In both acute and chronic infections, initial activation of CD8 T cells by cognate antigen presented by infected cells triggers a proliferative burst, massive production of inflammatory cytokines, and direct killing of infected host cells (Chung et al., 2021; McLane et al., 2019). This is accompanied by upregulation of the co‐inhibitory receptor PD‐1, a direct consequence of TCR signaling (Barber et al., 2006). In an acute infection, the virus eventually gets cleared, PD‐1 is downregulated, and most short‐lived effector cells (SLECs), which make up the majority of the antigen‐specific CD8 T cell pool at the peak of expansion, undergo apoptosis. At the same time, a pool of memory precursor cells (MPECs) is established that gives rise to long‐lived memory cells (Chung et al., 2021). These comprise effector memory, central memory, and tissue‐resident T cells that can persist in the absence of cognate antigen by relying on cytokines like IL‐7 and IL‐15 for survival (Becker et al., 2002; Schluns et al., 2000; Wherry & Ahmed, 2004). In the case of a reinfection with the same pathogen, memory cells are ready to mount a rapid response, leading to faster clearance of the virus. In contrast, chronic infections are not cleared, which leads to persistent TCR triggering, sustained expression of the co‐inhibitory receptor PD‐1, and inferior memory formation (Wherry et al., 2004). CD8 T cells differentiate toward exhaustion, characterized by sustained expression of co‐inhibitory receptors other than PD‐1 (e.g., CTLA‐4, Tim‐3, or TIGIT) and expression of the transcription factor TOX (Huang et al., 2025; McLane et al., 2019; Nieves‐Rosado et al., 2024; Scott et al., 2019; Wherry & Kurachi, 2015). In addition, exhausted virus‐specific CD8 T cells are restricted in effector cytokine production and in the expression of perforin and granzyme. These changes do not simply manifest transiently on the protein level but also are stably imprinted epigenetically and metabolically (Belk et al., 2022; Kissick & Ahmed, 2022; Park et al., 2023; Slater et al., 2026). Full commitment toward T cell exhaustion can therefore not easily be rewired by antigen removal, mimicking a resolved acute infection (Abdel‐Hakeem et al., 2021; Yates et al., 2021). T cell exhaustion is a gradual process, and in‐depth analysis of the virus‐specific CD8 T cell pool has revealed a heterogeneous landscape of cells that differ in their transcriptional profile and metabolism as well as location, which manifests in their differential contribution to controlling the infection. A stem‐like population (Tpex; TCF7^+^, CCR7^+^, PD‐1^+^, CXCR5^+^, CX3CR1^−^, CXCR6^−^) has been described, which resides in the white pulp of secondary lymphoid organs. Tpex do not directly contribute to virus control but serve as a reservoir (Broomfield & Groom, 2024; Utzschneider et al., 2016; Zehn et al., 2022) that feeds into the pool of effector‐like cells (TX‐eff; TCF7^−^, PD1^+^, CX3CR1^+^, CXCR6^−^) and ultimately into terminally exhausted cells (TX‐term; TCF1^−^, PD1^+^, CX3CR1^−^, CXCR6^+^) (Chen et al., 2019; Daniel et al., 2022; Lan et al., 2023; Sandu et al., 2020). TX‐eff and especially TX‐term exhibit reduced cytokine production and cytotoxicity compared to effector cells generated during acute infection, but they retain limited effector capacity and contribute to the partial viral control characteristic of chronic infection (McLane et al., 2019; Sandu et al., 2020; Shin & Wherry, 2007). Taken together, prior findings demonstrate that antigen‐specific CD8 T cells show distinct responses in acute and chronic infections, with responses starting similarly but diverging over time (Fig. 3).

**Figure 3:**
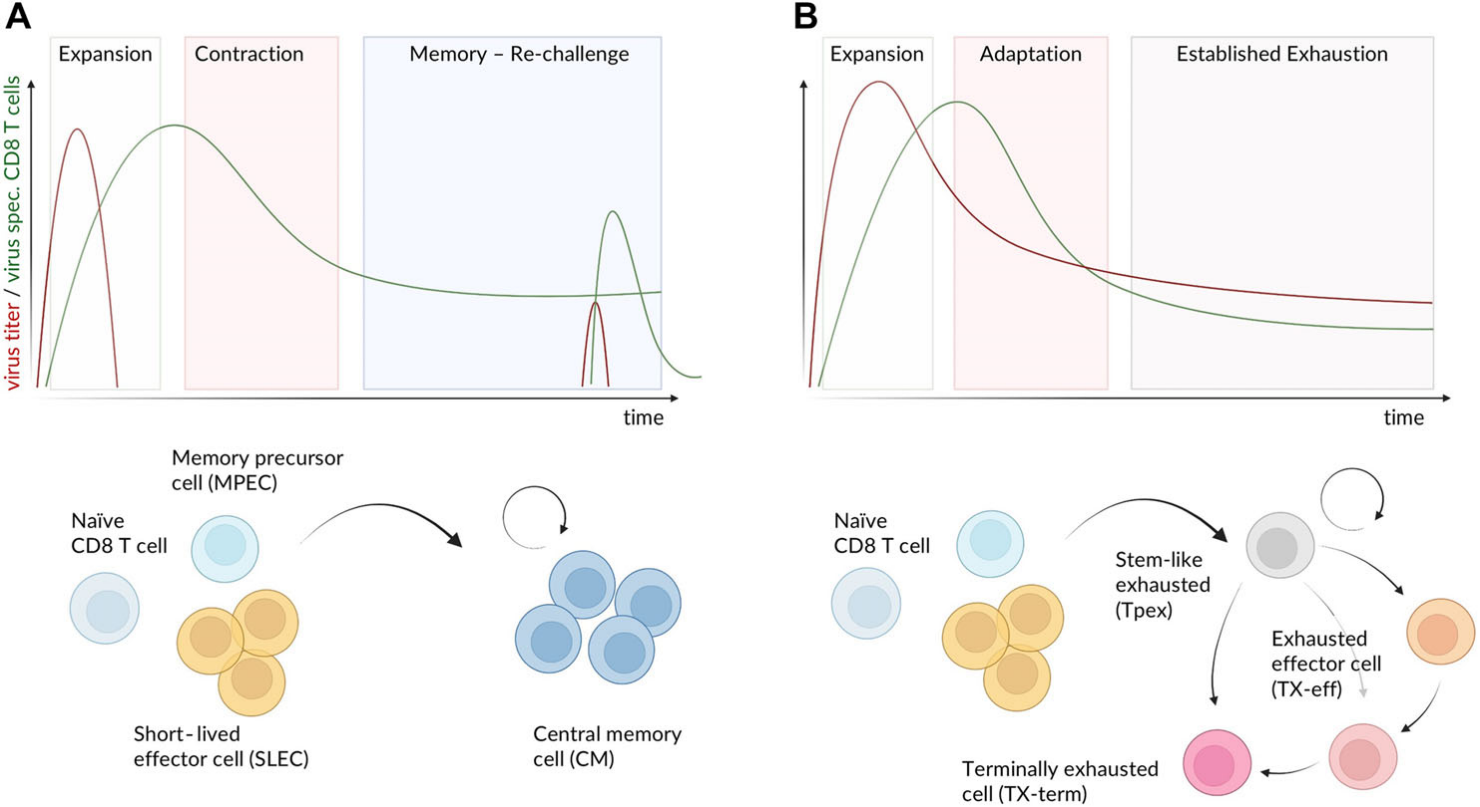
Virus‐specific CD8 T cell response to acute and chronic viral infections. (**A**) Graphical summary of antigen‐specific CD8 T cell priming, proliferation, and differentiation upon acute viral infection. Clearance of the pathogen will eventually allow central memory CD8 T cells to form, whereas short‐lived effector cells undergo apoptosis. (**B**) Graphical summary of CD8 T cell responses to chronic viral infection. Persistent antigen prevents memory formation and drives virus‑specific CD8 T cells toward exhaustion. Graphics were generated with BioRender.

How exactly CD8 T cells commit to an exhaustion trajectory; which transcription factors, cytokines, or metabolites drive or prevent exhaustion; and what niche components support the maintenance of stem‐like cells are all fundamental questions that remain only partially answered. Here, we aim to facilitate the ongoing efforts to answer these questions by providing a detailed experimental setup and analysis workflow.

The core of this article is the protocols used to perform the workflow needed to investigate CD8 T cells over the course of a chronic viral infection in mice. The basic protocols are graphically compiled in Figure 1 and include production of the virus strain of choice (Basic Protocol 1) and quantification of the viral titers of the viral stocks (Basic Protocol 2). This protocol can also be used to measure viral titers in tissues of an infected host animal (Alternate Protocol 1). To track LCMV‐specific CD8 T cells, peptide‐MHC‐I (pMHC‐I) tetramers can be used, which are discussed in Basic Protocol 3. Further, protocols are provided for the isolation of naïve CD8 T cells, which is primarily used to isolate CD8 T cells from the TCR‐transgenic mouse line P14 to perform adoptive cell transfer of virus antigen–specific cells (Basic Protocol 4 and Alternate Protocol 2). CD8 T cells isolated from the P14 mouse line are TCR monoclonal and specific to the immunodominant LCMV‐derived peptide gp33‐41. Lastly, following a guide on how to perform adoptive transfer of cells as well as the viral infection itself (Basic Protocol 5), we lay out protocols for organ harvest at the end point of the experiment and preparation of single‐cell suspensions from secondary lymphoid organs (Basic Protocol 7) and peripheral tissues (Alternate Protocol 3) as well as the staining and acquisition process for flow cytometric analysis (Basic Protocols 8 to 10). During the infection, animals must be closely monitored (Basic Protocol 6), which needs to be in accordance with animal experimentation law and the regulations of the responsible veterinary office.

*CAUTION*: LCMV is generally considered a Biosafety Level 2 (BSL‐2) pathogen. However, some countries classify LCMV as a Biosafety Level 3 (BSL‐3) pathogen, depending on the viral strain. Thus, appropriate guidelines and regulations for the use and handling of pathogenic microorganisms need to be followed. Further, LCMV is an airborne virus but can also be transmitted through saliva, urine, and feces. Infected mice are contagious and should never be co‐housed with mice that are not part of the same experimental procedure.

*NOTE*: All protocols involving animals must be reviewed and approved by the appropriate Animal Care and Use Committee and must follow regulations for the care and use of laboratory animals.

*NOTE*: All steps must be performed under a biosafety hood (Weisstechnik, BDK® Laminar Air Flow Ventilation Systems, or equivalent) unless stated otherwise.

## Basic Protocol 1 VIRUS PRODUCTION

This protocol outlines the production of LCMV in adherent mammalian cell cultures. We outline the steps required to efficiently propagate the virus and to generate high‐titer viral stocks suitable for downstream *in vitro* and *in vivo* applications. Careful attention should be paid to ensuring the viability and fitness of the cell line and to employing proper procedures and handling to maximize yield. The goal of this procedure is to obtain frozen LCMV stocks with high viral titers (1 × 10^7^ ffu/ml or higher). Use the same mother stock for virus production to avoid multiple passages, as this can lead to the emergence of new mutations that impact viral infection properties. An inoculum of 2 × 10^6^ ffu LCMV CL13 is widely used in the field and generally regarded as the standard dose for establishing chronic infection while minimizing the risk of an intermediate infection, which can lead to severe and potentially fatal immunopathology. Frozen viral stocks are generally stable for years when stored at −80°C, and re‐titration is only recommended if there is concern that storage conditions may have been compromised. Finally, it is standard practice to test newly produced and titrated viral stock batches *in vivo* in small cohorts before using them in scientific experiments.

**Time Considerations**

Virus production is a multiday effort. Culturing the cells prior to infection can take 1 week or longer depending on the desired amount of newly produced virus. The replating prior to infection takes about 1 to 2 hr for a considerable amount of stock production. On the next morning, plan 2 hr for the infection itself, including the 1‐hr incubation on the shaker. Virus harvests are done 48 hr and 72 hr post‐infection (hpi). Harvest of the virus needs to be done swiftly. Try to minimize the time between supernatant harvest and freezing. Only harvest as many flasks per batch as can be processed within a short period of time, preferably <30 min. Working in teams and processing a maximum of 6 to 8 flasks per batch is recommended.

### Materials

Baby hamster kidney cells (BHK‐21 cells; ATCC, cat. no. CCL‐10)
Maintenance medium (see recipe), 37°C
Phosphate‐buffered saline (PBS; BioConcept, cat. no. 3‐05F29‐I)
Trypsin (Trypsin 0.05% / EDTA 0.02% in DPBS, w/o: Ca and Mg, PAN Biotech, cat. no. P10‐023100)
Trypan blue (0.4%; Sigma‐Aldrich, cat. no. T8154; optional)
Primary virus stocks, mycoplasma‐tested (LCMV Arm, WE, CL13, Doc; can be purchased from EVA, ATCC, or others or provided by academic labs)
Infection medium (see recipe), 37°C

T150 cell culture flasks (TPP, cat. no. 90150)
50‐ml centrifuge tubes (TPP, cat. no. 91050)
Standard tabletop centrifuge (Eppendorf AG, model no. 5910 Ri, or equivalent), 4°C and room temperature
Automated cell counter (Countess, Thermo Fisher Scientific, or equivalent) or hemocytometer
Microscope (Olympus, cat. no. CKX41‐B, or equivalent; optional)
Shaker (Heidolph Duomax 1030, cat. no. 543‐32205‐00)
Glass bottles (Duran, cat. no. 218014459, or equivalent), pre‐chilled on ice
Reservoirs (Corning, cat. no. 4870)
Pre‐labeled cryotubes (Sarstedt, cat. no. 72.379.007, or similar)

*NOTE*: All solutions and equipment coming into contact with cells must be sterile, and proper sterile technique should be used accordingly, including use of a laminar flow cabinet (Weisstechnik, BDK® Laminar Air Flow Ventilation Systems, or equivalent).

*NOTE*: Unless otherwise indicated, keep the cell culture flasks in a cell culture incubator (Heracell^TM^, Thermo Fisher Scientific, cat. no. 50116047, or equivalent) at 37°C, 5% CO_2_, and 85% to 95% humidity.

1 Expand the BHK‐21 cells in T150 cell culture flasks in 30 ml maintenance medium per flask.
  Use the cell line that matches the viral strain of interest for production (Table 1).
2 Split cells 1:10 when they are 90% to 100% confluent (every 3 to 4 days).
3 Remove the medium and wash once with 10 ml PBS.
4 Add 3 to 4 ml trypsin for 5 min at 37°C to detach the cells.
5 Add up to 5 ml fresh medium to inactivate the trypsin. Harvest the cell suspension in 50‐ml centrifuge tubes.
6 Centrifuge the cell suspension for 5 min at 550 × *g* and discard the supernatant.
7 Resuspend the cells and count the cells with an automated cell counter or trypan blue, a hemocytometer, and a microscope. Then, replate 10^8^ cells or ~80% and allow them to reattach.
  This takes roughly 2 to 3 hr. Cells should reach ∼80% confluence at the time of infection for maximum virus yield.
  BHK‐21 cells tend to need longer for proper reattachment. Standard procedure for BHK‐21 cells is to split them to 80% confluence on the evening before the viral infection and to start with the infection on the following morning
8 Dilute the primary virus stock in infection medium to achieve a multiplicity of infection (MOI) of 0.01 in a total volume of 5 ml infection medium per cell culture flask for harvest at 48 hpi. Alternatively, use an MOI of 0.1 for a first harvest at 24 hpi.
  The MOI indicates the relation between infectious particles and cells. An MOI of 0.1 therefore means that 1 virion is added per every 10 cells.
9 Distribute 5 ml of the virus dilution to each flask to infect the cells.
10 Incubate the cell culture flasks for 1 hr at room temperature on a shaker set to a low speed.
  This ensures optimal distribution of the virus diluent on the cells and increases overall virus titers at the day of harvest.
11 Add 15 ml maintenance medium and incubate at 37°C in the cell culture incubator.
  Steps 11 to 17 need to be done as fast as possible. LCMV is not very stable at room temperature, so working efficiently is crucial. Depending on the number of flasks to process, it is highly recommended to work in a team.
12 After 48 hr, collect the supernatant in 50‐ml centrifuge tubes and add 20 ml fresh maintenance medium to the cells.
13 Incubate flasks for another 24 hr.
14 Centrifuge the supernatant from step 12 for 5 min at 550 × *g*, 4°C, immediately after harvest.
15 Decant supernatant of all centrifuge tubes into a pre‐chilled glass bottle (on ice) and quickly swirl the bottle to guarantee a homogeneously mixed solution.
16 Pour the supernatant into a reservoir and aliquot into pre‐labeled cryotubes.
  Use a repeater pipet (Eppendorf, cat. no. 4982000012) if available; it will allow for faster aliquoting of the virus stocks. Aliquots of 1 ml are recommended, which is enough to infect five animals with 200 µl each. Do not refreeze virus stocks.
17 Immediately freeze and store new virus stocks at –80°C.
18 Repeat steps 11 to 17 on the next day (72‐hr stock production). Do not add fresh medium after supernatant harvest but rather discard the cell culture flasks.
19 Proceed with determining the viral titers of the 48‐hr and 72‐hr stocks using a focus‐forming assay (FFA; see Basic Protocol 2).

## Basic Protocol 2 VIRUS TITER QUANTIFICATION FROM VIRUS STOCKS BY FOCUS‐FORMING ASSAY

The FFA is a sensitive and reliable method for titrating viral stocks by counting discrete foci of infected cells. Viral titers are expressed as focus‐forming units (ffu) per volume. Because LCMV is non‐cytopathic, infected cells do not produce visible plaques of lysed cells. Instead, detection relies on immunostaining of intracellular viral proteins. The FFA provides a straightforward approach to quantify infectious particles. The use of fresh reagents, viable and healthy host cells, and proper handling is critical to ensure efficient staining and accurate quantification of viral particles.

**Time Considerations**

The FFA takes roughly 3 days, including an incubation period of 2 days. Plan for 3 to 4 hr on the first day, especially if organs are analyzed. Factor in more time if the organs must be harvested on the day of the FFA. The staining and development of the assay are done 2 days after inoculation and will take ∼4 hr.

### Materials

MC57G cells (adherent murine fibroblast cell line; ATCC, cat. no. CRL‐2295), in maintenance medium (see recipe)
PBS (BioConcept, cat. no. 3‐05F29‐I)
Trypsin (Trypsin 0.05% / EDTA 0.02% in DPBS, w/o: Ca and Mg, PAN Biotech, cat. no. P10‐023100)
Infection medium (see recipe), 37°C
Trypan blue (0.4%; Sigma‐Aldrich, cat. no. T8154; optional)
Virus samples of interest (see Basic Protocol 1)
Overlay solution (see recipe)
4% (w/v) formaldehyde (MeOH free, 10% ultrapure; Polysciences, cat. no. 04018) in PBS (BioConcept, cat. no. 3‐05F29‐I)
1% (v/v) Triton‐X‐100 solution
10% (v/v) fetal bovine serum (FBS; Life Technologies, cat. no. 10270106) in PBS (BioConcept, cat. no. 3‐05F29‐I) (optional)
Rat anti‐LCMV‐NP monoclonal antibody (produced in‐house or purchased, e.g., clone VL‐4, Merck, cat. no. MABF2781‐100UL) diluted in 1% (v/v) FBS (Life Technologies, cat. no. 10270106) in PBS (BioConcept, cat. no. 3‐05F29‐I)
Detection antibody (Peroxidase AffiniPure Goat Anti‐Rat IgG (H+L), Jackson ImmunoResearch Laboratories, cat. no. 112‐035‐003) diluted 1:400 in 1% (v/v) FBS (Life Technologies, cat. no. 10270106) in PBS (BioConcept, cat. no. 3‐05F29‐I)
Ortho‐phenylenediamine (OPD) tablet (20 mg, used per 50 ml detection solution; Sigma‐Aldrich Chemie, cat. no. P3888)
Detection Buffer A: 0.2 M Na_2_HPO_4_•2H_2_O (Sigma‐Aldrich Chemie, cat. no. S3264) in double‐distilled water (ddH_2_O) (stored at room temperature)
Detection Buffer B: 0.1 M citric acid (Sigma‐Aldrich Chemie, cat. no. 251275) in ddH_2_O (stored at 4°C)
ddH_2_O
Hydrogen peroxide (H_2_O_2_), 35% (Thermo Fisher Scientific, cat. no. 202465000)

50‐ml centrifuge tubes (TPP, cat. no. 91050)
Standard tabletop centrifuge (Eppendorf AG, model no. Ri, or equivalent)
Automated cell counter (Countess, Thermo Fisher Scientific, or equivalent) or hemocytometer
Microscope (Olympus, cat. no. CKX41‐B, or equivalent; optional)
Reservoirs (Corning, cat. no. 4870)
Round‐bottom 96‐well plates (TPP, cat. no. 92097)
Multichannel pipet (Eppendorf)
24‐well culture plates (TPP, cat. no. 92024)
Virus waste container
10‐ml serological pipets

*NOTE*: All solutions and equipment coming into contact with cells must be sterile, and proper sterile technique should be used accordingly, including use of a laminar flow cabinet (Weisstechnik, BDK® Laminar Air Flow Ventilation Systems, or equivalent).

*NOTE*: Unless otherwise indicated, keep the cell culture plates in a cell culture incubator (Heracell^TM^, Thermo Fisher Scientific, cat. no. 50116047, or equivalent) at 37°C, 5% CO_2_, and 85% to 95% humidity.

#### Cell harvest

1 Remove the cell culture medium (maintenance medium) from the MC57G cells and wash once with 10 ml PBS.
  It is recommended to harvest MC57G cells when they have formed a confluent monolayer. MC57G cells are cultured in T150 cell culture flasks (TPP, cat. no. 90150) until the day of use. One confluent T150 flask yields sufficient cells for approximately five 24‐well culture plates. Four virus titrations can be done per 24‐well culture plate. It is recommended to quantify the viral titers of virus stocks or infected tissues in technical duplicates.
2 Add 3 to 4 ml trypsin for 5 min at 37°C to detach the cells.
3 Add up to 5 ml fresh infection medium to neutralize the trypsin and harvest the cell suspension in 50‐ml centrifuge tubes.
4 Centrifuge the cell suspension for 5 min at 550 × *g* and discard the supernatant.
5 Resuspend the cell pellet in 2 ml fresh infection medium.
6 Count the cells with an automated cell counter or trypan blue, a hemocytometer, and a microscope and adjust the concentration to 0.8 × 10^6^ cells/ml in infection medium.
7 Prepare 6 ml cell suspension per 24‐well culture plate. Keep the cells on ice while virus samples of interest are thawed and diluted (see steps 8 to 10).

#### Virus sample preparation

8 Pour the required volume of infection medium into a reservoir and prepare round‐bottom 96‐well plates by adding 130 µl infection medium to columns 2 to 11 using a multichannel pipet.
9 Add 200 µl of each virus to column 1 in duplicates.
  Preparing duplicates is important for multichannel pipetting afterward (see step 11).
10 Perform a serial dilution using a multichannel pipet: transfer 60 µl from the first row to the second row, mix thoroughly by pipetting up and down, and continue this process sequentially. Replace pipet tips every 3 to 4 rows to minimize technical bias.

#### Virus adsorption

11 Pour the required volume of cell suspension into a reservoir and dispense 200 µl MC57G cell suspension (see step 7) into each well of a 24‐well culture plate (100 µl × 2 using a multichannel pipet). Gently tap the plate to ensure uniform distribution.
  Resuspend the cells if they do not evenly distribute and are concentrated at the edges.
12 Add 200 µl of the virus dilution series (see step 10) from columns 11, 9, 7, 5, 3 and 1 to the cells. Proceed from the highest to the lowest dilution to avoid having to change pipet tips.
13 After gently mixing by moving the plate in a figure‐eight motion on the bench top, incubate the plates 2 to 4 hr at 37°C. Use a microscope to confirm that the cells attached to the cell culture plate's bottom before proceeding to step 14.
14 Add approximately 150 to 200 µl overlay solution per well.
  The overlay solution is a very viscous solution. Therefore, it is inconvenient to handle it with pipet tips; use 10‐ or 25‐ml serological pipets instead. Prioritize efficiency over accuracy in this step.
15 Incubate plates for 2 days at 37°C.

#### Staining

16 Proceed with staining only if the cell monolayer is 100% confluent.
  Should the cells not have reached confluence, incubation can be extended by up to 24 hr. Avoid letting the wells dry out during the staining procedure until the cells are fixed. If many plates are processed simultaneously, work in a team or stain the plates in batches.
17 Gently discard the overlay into a designated virus waste container.
18 Add 3 to 4 drops of 4% formaldehyde to each well using a 10‐ml serological pipet.
  Avoid pipetting directly onto the cell monolayer, as this may disrupt the attached cells. Instead, slightly tilt the plate and dispense the liquid along the edge of the well to minimize the risk of cell detachment.
19 Incubate 30 min at room temperature.
20 Discard the 4% formaldehyde into the virus waste container and wash once with PBS.
21 Add 3 to 4 drops of 1% Triton‐X‐100 solution using a 10‐ml serological pipet.
22 Incubate 20 min at room temperature.
23 Discard the Triton‐X‐100 solution, wash once with PBS, and then optionally add 200 µl of 10% FBS in PBS to block nonspecific binding.
24 Incubate 45 to 60 min at room temperature.
25 Discard the blocking solution into the virus waste container and wash once with PBS.
26 Add 200 µl primary antibody (rat anti‐LCMV‐NP monoclonal antibody) diluted in 1% FBS in PBS.
  The primary antibody we use is produced in‐house, and each batch is titrated individually. The reagent can be purchased from different vendors, and it is highly recommended to titrate it prior to experimental use.
27 Incubate 60 min at room temperature.
28 Discard the primary solution and wash 1 to 2 times with PBS.
29 Add 200 µl detection antibody diluted 1:400 in 1% FBS in PBS.
30 Incubate for 60 min at room temperature.
31 During the incubation time, prepare the detection solution: dissolve one OPD tablet (20 mg) in 12.5 ml Detection Buffer A. Once fully dissolved, add 12.5 ml Detection Buffer B and 25 ml ddH_2_O.
  The OPD tablet should be fully dissolved after 20 min in Detection Buffer A at room temperature.
32 Discard the detection antibody solution from step 30 and wash wells twice with PBS.
33 Add 50 µl H_2_O_2_ to the detection solution prepared in step 31 immediately prior to use.
34 Add 200 µl of the finalized detection solution to each well.
35 Incubate for ∼30 min or until clearly detectable foci have appeared.
  Depending on the quality of the buffers, the duration of visible foci development can differ. Optimally, foci appear after several minutes.
36 Discard the detection solution and wash the plates with tap water.
37 Air‐dry the plates or directly proceed with counting of the foci.
  There is no rush in quantifying the foci, as the staining is stable for years.

## Alternate Protocol 1 VIRUS TITER QUANTIFICATION FROM INFECTED TISSUES BY FOCUS‐FORMING ASSAY

Virus titers can also be measured from infected tissues, and not only from purified virus stocks (Basic Protocol 1). Therefore, this protocol explains the harvest and preparation of organs for this purpose.

**Time Considerations**

Harvested organs do not need to be analyzed immediately and can be frozen. Thawing and lysing organs should be quick and not exceed 15 to 20 min. If more organs need to be analyzed, work in batches. The duration of thawing and lysis before plating organ lysate should be minimized.

### Additional Materials (also see Basic Protocol 2)

Virus‐infected murine organs (e.g., from Basic Protocol 5)

2‐ml microtubes (Sarstedt, cat. no. 72.695.500)
Ball‐bearing beads (Berani Kugellager, cat. no. KU.5 NIRO 40, or equivalent)
TissueLyser (Qiagen, cat. no. 9003240) or equivalent
Microcentrifuge (Thermo Fisher Scientific, cat. no. 75002402), 4°C

Additional reagents and equipment for organ harvesting

1 Prepare 2‐ml microtubes with 1 ml infection medium each and add 1 ball‐bearing bead per tube.
2 Collect virus‐infected murine organs separately in the prepared microcentrifuge tubes.
  Samples may be frozen and stored at –80°C or processed immediately for FFA.
3 Perform steps 1 to 7 of Basic Protocol 2.
  It is important to minimize the time between thawing the virus‐containing samples and adding them to the cell culture. Therefore, it makes sense to have the cells ready before homogenization of the organs. Keep the cells on ice and only plate them once the infected organs are homogenized and centrifuged (see steps 4 and 5).
4 Prior to starting the FFA, to release the virus from the infected cells, homogenize the organs for 90 s at 25 Hz in a TissueLyser or equivalent.
  Homogenized organs can be stored at –80°C or processed immediately.
  It is not recommended to freeze intact organs, thaw them for homogenization, and then re‐freeze them; the organs should ideally be frozen and thawed only once.
5 Centrifuge the samples in a pre‐cooled microcentrifuge for 5 min at 3500 × *g*, 4°C.
  During the spinning time, it is recommended to thaw a virus stock that can then serve as a positive control for the assay.
6 Continue from step 8 of Basic Protocol 2, but instead of using pure virus stocks, dilute the supernatant of the infected organs and add it to the cells.

#### Understanding Results

In theory, the number of foci forming in the wells should follow the pattern of the serial dilution, which is performed 1:10. However, the readout is often nonlinear, which can be for several reasons. A very low foci count (in the range of 0 to 10 per well) as well as a very high foci count (saturating the wells’ surface) should not be used for viral titer calculation. It is recommended to choose a dilution that yields between 20 and 60 foci, ensuring measurements are within the linear range of the assay.

## Basic Protocol 3 PEPTIDE‐MHC‐I TETRAMER PRODUCTION

CD8 T cells’ specificities in a non‐transgenic mouse are very heterogeneous. To detect endogenous LCMV‐specific CD8 T cells, pMHC‐I tetramers can be used. Alternatively, TCR‐transgenic P14 CD8 T cells can be adoptively transferred (Basic Protocol 5) and distinguished from the host animals’ cells by congenic markers (e.g., CD45.1 and CD45.2). The current and the following protocols explain how to implement these two options.

Production of pMHC‐I monomers, the first step in generating tetramers for antigen‐specific CD8 T cell detection, is a multistep process that is not provided here. MHC‐I monomers can either be purchased (e.g., from the NIH Tetramer Core Facility at Emory University; NIH Tetramer Core Facility at Emory University, n.d.‐b.) or produced in‐house following a published workflow (NIH Tetramer Core Facility at Emory University, n.d.‐a.). It is important to obtain biotinylated monomers for tetramerization using fluorochrome‐conjugated streptavidin. Monomers should be stored at –80°C until tetramerization. A list of antigenic peptides in combination with the respective MHC‐I allele to detect LCMV‐specific CD8 T cells is given in Table 2. The fate of LCMV‐specific CD8 T cells varies according to their antigen specificity. Although the three major immunodominant epitopes NP396, GP33, and GP276 lead to both a strong effector response and memory development during an acute infection, with NP396‐ and GP33‐specific CD8 T cells detectable at higher levels than GP276‐specific CD8 T cells, this changes in a chronic setting. In particular, GP33‐specific CD8 T cells stay detectable at high levels and show all subpopulations of exhaustion, whereas NP396‐specific CD8 T cells nearly disappear and reflect a much more terminally exhausted fate. GP276‐specific CD8 T cells acquire a fate in between (Klein et al., 2023; Wherry et al., 2003). Depending on the scientific question, it is crucial to consider which specificity to focus on. The tetramerization process is identical for all epitopes and is explained below.

**Table 2:** pMHC‐I Constructs Used to Detect LCMV Antigen–Specific CD8 T Cells

Name	Peptide sequence	Allele restriction
LCMV NP 396‐404	FQPQNGQFI	H2‐Db
LCMV GP33‐41	KAVYNFATC	H2‐Db
LCMV GP33‐41 (C41M)	KAVYNFATM	H2‐Db
LCMV GP276‐286	SGVENPGGYCL	H2‐Db
LCMV NP 118‐126	RPQASGVYM	H2‐Ld*^a^*

^*a*^ H2‐Ld is present in BALB/C mice but not in C57BL/6.

**Time Considerations**

The whole process takes 8 hr, with 1 min of hands‐on work every 2 hr.

### Materials

Biotinylated pMHC‐I monomer (e.g., gp33H2‐Db, NIH Tetramer Core Facility, Emory University)
Fluorochrome‐conjugated streptavidin (e.g., APC‐Streptavidin, BioLegend, cat. no. 405243)

Rotating mixer or tumbling shaker (Labgene Scientific, cat. no. R5010E, or equivalent)

1 Thaw a biotinylated pMHC‐I monomer (e.g., gp33‐H2Db) and keep it at 4°C during tetramerization (see steps 2 to 4).
2 To tetramerize the monomers, add a fluorochrome‐conjugated streptavidin reagent. Incubate for 2 hr.
  The exact procedure depends on the amount or concentration of the monomer and on the concentration of the conjugated streptavidin. This protocol is based on monomer aliquots of 100 µg in 50 µl (2 mg/ml) and a concentration of the conjugated streptavidin of 1 mg/ml.
  For APC‐streptavidin, add 5 × 4 µg in 5 × 20 µl every 2 hr.
3 During the 2‐hr incubation time, protect the mixture from light and ensure slow but constant movement of the reaction fluid (e.g., use a rotating mixer or tumbling shaker).
4 Store the newly tetramerized reagent at 4°C, protected from light, until use.
  The reagent is stable for several weeks.
  Use the reagent together with the fluorochrome‐conjugated antibodies during the surface staining procedure (see Basic Protocol 8).
  The optimal concentration of the reagent varies from batch to batch and is influenced by the fluorophore‐conjugated streptavidin as well as by the peptide. Therefore, best practice includes titration of the reagent before use.

#### Understanding Results

See Table 3 and Figures 4A and 4B. Include tetramer staining if endogenous pMHC‐I‐specific CD8 T cells are of interest. As shown in Figure 4A, fluorescently labeled gp33MHC‐I tetramers bind very specifically, with staining exclusive to gp33MHC‐I‐specific P14 CD8 T cells when mixed with C57BL/6 wild‐type (WT) splenocytes. Not all pMHC‐I tetramers show the same quality of staining. It is therefore recommended to choose a bright fluorochrome to tetramerize a poorly staining pMHC‐I, monomer and vice versa. It is also possible to combine multiple pMHC‐I tetramers if more than one TCR specificity is of interest.

**Table 3:** Flow Cytometry Panel to Test Quality of gp33MHC‐I Tetramers

Fluorochrome/dye	Antigen	Clone	Concentration (mg/ml)	Working dilution	Catalog number	Vendor
BUV395	CD8a	53‐6.7	0.2	1:400	563786	BD Biosciences
BUV737	CD45.2 (Ly5.2)	104	0.2	1:200	612778	BD Biosciences
BV421	TCR Vα2	B20.1	0.2	1:1600	127825	BioLegend
BV785	CD45.1 (Ly5.1)	A20	0.2	1:400	110743	BioLegend
PE‐Cy5	CD45R/B220	RA3‐6B2	0.2	1:1600	103210	BioLegend
APC	gp33H2‐Db tetramer	Prepared in‐house	N/A	1:100	N/A	N/A
LIVE/DEAD™ Fixable Near‐IR Dead Cell Stain Kit	N/A	N/A	N/A	1:500*^a^*	L34976	Thermo Fisher Scientific

^*a*^ This dilution is after reconstitution with 50 µl dimethyl sulfoxide (DMSO).

**Figure 4:**
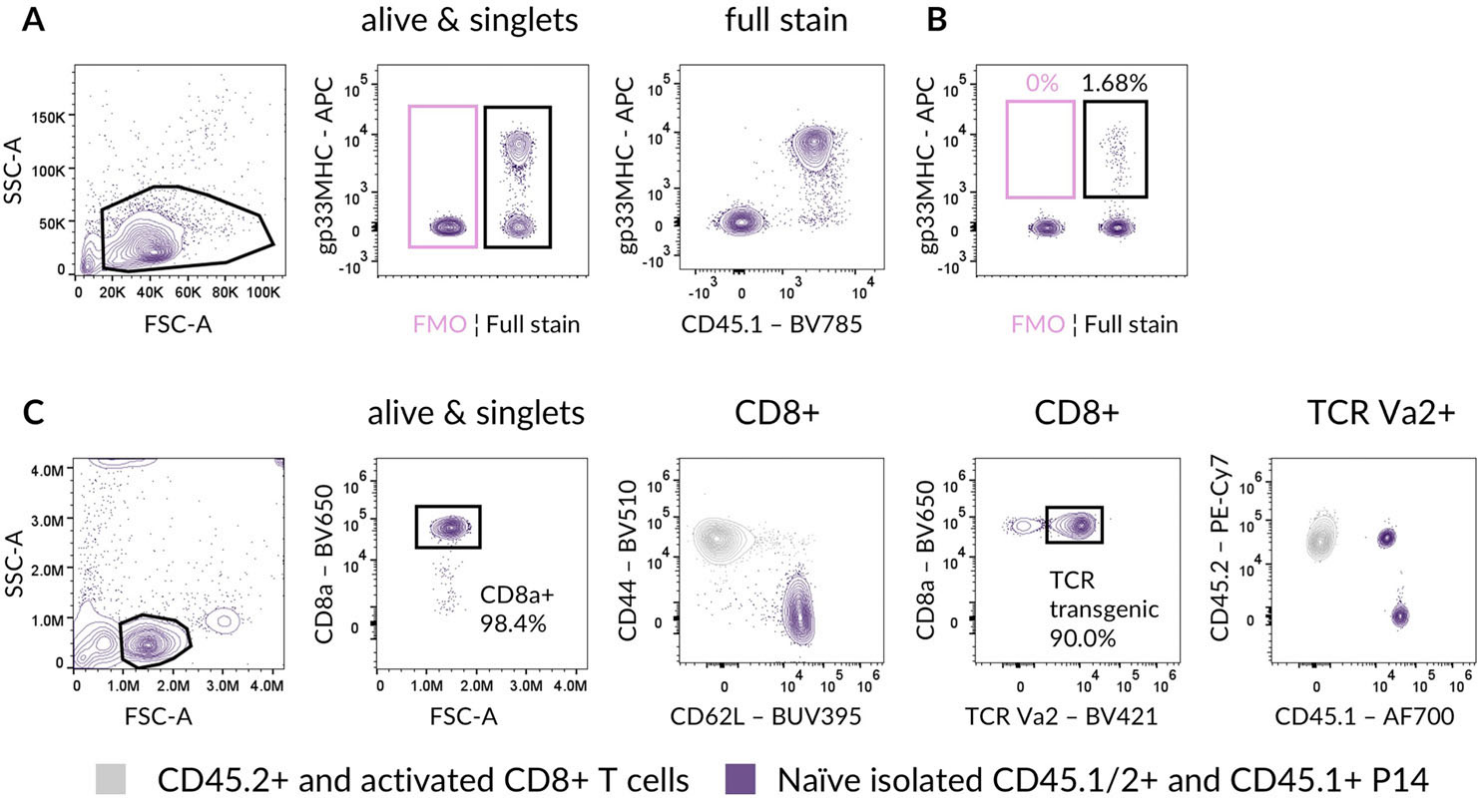
Quality control of pMHC‐I tetramer staining and analysis of freshly isolated naïve TCR‐transgenic P14 CD8 T cells. (**A**) Gating strategy for verifying the specific binding of the produced gp33MHC‐I tetramer to gp33MHC‐I‐specific CD8 T cells. A mix of C57BL/6 wild‐type (WT) (CD45.2^+^) and TCR‐transgenic (CD45.1^+^) lymphocytes were stained. After gating on live cells and singlets (data not shown), comparison of a fluorescence‐minus‐one (FMO) control to the fully stained sample shows a clear signal only in the fully stained sample. Investigating only the fully stained sample, gp33MHC‐I tetramer–positive cells are exclusively present in the CD45.1^+^ fraction, which are the gp33‐41 peptide–specific TCR‐transgenic CD8 T cells (P14). (**B**) Staining of C57BL/6 WT splenocytes isolated from a chronically infected mouse (21 dpi) reveals a small but distinct fraction of LCMV peptide gp33‐41–specific CD8 T cells. (**C**) Staining of freshly isolated splenocytes using the naïve CD8 T cell negative selection kit from StemCell Technologies™. Gating on live and single cells (data not shown) shows isolation purity. Comparison of the isolated CD8 T cells (deep purple) with activated C57BL/6 splenocytes (gray) shows that only naïve (CD62L^+^ / CD44^−^) CD8 T cells were isolated. Staining for the TCR alpha chain (TCR Vα2) of the P14 TCR confirms the TCR‐transgenic genotype because the fraction of TCR Vα2^+^ among CD8 T cells in C57BL/6 WT is only ∼10% (data not shown). Here, naïve CD8 T cells were isolated from two mice that differ in their congenic marker (CD45.1 homozygous and CD45.1/2 heterozygous). Staining a mixture of CD8 T cell isolates with the respective congenic markers showcases the clear distinction between the two populations, enabling double transfer experiments, if required.

## Basic Protocol 4 NAÏVE CD8 T CELL ISOLATION USING StemCell^TM^ KIT

To isolate naïve CD8 T cells from secondary lymphoid organs of mice, the negative selection kit from StemCell Technologies™ can be used. This protocol follows the official guidelines from the product's vendor. A naïve CD8 T cell purity of up to 98% can be achieved. Murine spleens and lymph nodes are used as a source for naïve CD8 T cells. The isolated naïve CD8 T cells can be used for adoptive transfer into recipient mice (Basic Protocol 5), analysis by flow cytometry (Basic Protocols 8 to 10), or cell culture. For the isolation of bulk CD8 T cells, the MojoSort™ Mouse CD8 T Cell Isolation Kit can be used as an alternative (Alternate Protocol 2), which allows an isolation purity of up to 90%.

**Time Considerations**

Both this protocol and Alternate Protocol 2 require ∼90 min from organ harvest to completion of the cell isolation.

### Materials

Mice (e.g., from B57BL/6 or P14 mouse line)
MACS buffer (see recipe), 4°C
EasySep™ Mouse Naïve CD8+ T Cell Isolation Kit (StemCell Technologies, cat. no. 19858), including FcR blocker, isolation antibody cocktail, and streptavidin RapidSpheres
Desired buffer or cell culture medium depending on downstream experiment
Trypan blue (0.4%; Sigma‐Aldrich, cat. no. T8154; optional)
PBS, sterile (BioConcept, cat. no. 3‐05F29‐I; optional)

2‐ml microtubes (Sarstedt, cat. no. 72.695.500)
70‐µm cell strainer (SmartStrainer, Miltenyi Biotec, cat. no. 130‐098‐462)
50‐ml centrifuge tubes (TPP, cat. no. 91050)
Standard tabletop centrifuge (Eppendorf AG, model no. 5910 Ri, or equivalent), room temperature and 4°C
Vortex
FACS tubes (5‐ml polystyrene round‐bottom tubes, Corning, cat. no. 352054)
StemCell^TM^ magnet (StemCell Technologies, cat. no. 18103)
Automated cell counter (Countess, Thermo Fisher Scientific, or equivalent) or hemocytometer
Microscope (Olympus, cat. no. CKX41‐B, or equivalent; optional)

Additional reagents and equipment for mouse euthanasia and organ harvest

NOTE: Unless stated otherwise, all procedures must be done under sterile conditions and at 4°C or on ice.

1 Euthanize mice and harvest murine spleen and lymph nodes into 2‐ml microtubes containing ice‐cold MACS buffer.
  If you are planning to adoptively transfer LCMV‐specific CD8 T cells (see Basic Protocol 5), use spleens and lymph nodes from P14 transgenic mice.
  The organs can be kept on ice for several hours without significant loss of viable cells. Nevertheless, it is beneficial to process the organs as soon as possible.
2 Pass the spleen and lymph nodes through a 70‐µm cell strainer placed on a 50‐ml centrifuge tube.
  You can use the flat part of a syringe (e.g., Terumo, cat. no. MDSS01SE) plunger to pass the organ through the strainer.
3 Rinse the cell strainer with 10 ml cold MACS buffer.
4 Centrifuge the cell suspension for 5 min at 550 × *g*, 4°C, and discard the supernatant by pouring it off carefully.
5 Resuspend the cell pellet in a total volume of 500 µl cold MACS buffer and remove tissue clumps with a pipet tip.
6 Add 5 µl FcR blocker and 25 µl isolation antibody cocktail (both from the EasySep™ Mouse Naïve CD8+ T Cell Isolation Kit) to the cell suspension.
7 Gently mix the cells by pipetting.
8 Incubate at room temperature for 10 min.
9 Vortex streptavidin RapidSpheres (from the EasySep™ Mouse Naïve CD8+ T Cell Isolation Kit) for 30 s prior to use. Add 50 µl streptavidin RapidSpheres to the cell suspension and gently mix by pipetting.
10 Incubate at room temperature for 5 min.
11 Transfer the cell suspension to an empty FACS tube.
12 Rinse the centrifuge tube with 2 ml cold MACS buffer and transfer this buffer to the FACS tube to maximize the yield.
13 Place the FACS tube into the StemCell^TM^ magnet for 5 min.
14 Transfer the non‐magnet‐bound fraction to a fresh 50‐ml centrifuge tube containing 10 ml cold MACS buffer.
  It is critical to not disturb the magnet‐bound fraction of the cells with the pipet tip while harvesting the non‐bound cell fraction.
15 Centrifuge 5 min at 550 × *g* and discard the supernatant.
16 Resuspend the cell pellet in the desired buffer or cell culture medium depending on the downstream experiment.
17 Count the cells using an automated cell counter or trypan blue, a hemocytometer, and a microscope.
  Take an aliquot of the isolated naïve CD8 T cells and analyze the purity and the transgenic and/or congenic information of the isolated cells by flow cytometry (see Basic Protocol 8).
  At this point, the cells are ready for further processing and analyses.
18 For flow cytometric analysis, pellet the desired number of cells and proceed with the staining (see Basic Protocol 8). For adoptive transfer into recipient mice, dilute the cells in sterile PBS and keep them on ice until intravenous injection (see Basic Protocol 5).

## Alternate Protocol 2 NAÏVE CD8 T CELL ENRICHMENT USING MojoSort^TM^ KIT

For the isolation of bulk CD8 T cells, the MojoSort™ Mouse CD8 T Cell Isolation Kit can be used as an alternative to the naïve CD8 T cell isolation described in Basic Protocol 4. Alternate Protocol 2 allows for an enrichment purity of up to 90%.

**Time Considerations**

Both Basic Protocol 4 and Alternate Protocol 2 require ∼90 min from organ harvest to completion of the cell isolation.

### Additional Materials (also see Basic Protocol 4)

MojoSort™ Mouse CD8 T Cell Isolation Kit (BioLegend, cat. no. 480008), including streptavidin nanobeads
MACS buffer (see recipe)

*NOTE*: Unless stated otherwise, all procedures must be done under sterile conditions and at 4°C or on ice.

1 Follow Basic Protocol 4, steps 1 to 5.
2 Add 5 µl FcR blocker and 10 µl isolation antibody cocktail to the cell suspension (both from the MojoSort™ Mouse CD8 T Cell Isolation Kit).
3 Gently mix the cell suspension by pipetting.
4 Incubate the cells for 15 min on ice.
5 Vortex streptavidin nanobeads (from MojoSort™ Mouse CD8 T Cell Isolation Kit) for 30 s prior to use. Add 10 µl streptavidin nanobeads to the cell suspension and gently mix by pipetting.
6 Incubate for 15 min on ice.
7 Transfer the cell suspension to a FACS tube and add 2 ml MACS buffer.
8 Place the FACS tube into the StemCell^TM^ magnet for 5 min.
9 Transfer the non‐magnet‐bound fraction to a fresh FACS tube containing 1 ml MACS buffer.
  It is critical to not disturb the magnet‐bound fraction of cells while harvesting the non‐bound cell fraction.
10 Centrifuge 5 min at 550 × *g* and discard the supernatant.
11 Follow Basic Protocol 4, steps 16 to 18.

#### Understanding Results

See Table 4 and Figure 4C. It is highly recommended to check the quality of the CD8 T cell isolation. Naive CD8 T cells will be negative for the activation markers CD44 and CD69 (not shown) but positive for CD62L. If the isolated cells will be adoptively transferred, it is important to check if the congenic marker is different from the one of the hosts. In the depicted example in Figure 4C, the combination of the congenic markers CD45.1 and CD45.2 is used to showcase that three different populations of cells (CD45.1 homozygous / CD45.2 homozygous / CD45.1/2 heterozygous) can be distinguished. This allows for adoptive transfer of up to two different donor cell populations. Additional genotyping can be done to confirm the quality of the isolated cell pool. Here, TCR Va2 was used as a marker to confirm the LCMV‐specific TCR P14.

**Table 4:** Flow Cytometry Panel to Assess Purity and Phenotype of Isolated Naïve CD8 T Cells

Fluorochrome/dye	Antigen	Clone	Concentration (mg/ml)	Working dilution	Catalog number	Vendor
BUV395	CD62L (L‐selectin)	MEL‐14	0.2	1:1600	569400	BD Biosciences
BV421	TCR Vα2	B20.1	0.2	1:1600	127825	BioLegend
BV510	CD44	IM7	0.2	1:400	103044	BioLegend
BV650	CD8a	53‐6.7	0.2	1:1600	100742	BioLegend
PE‐Cy7	CD45.2 (Ly5.2)	104	0.2	1:400	109830	BioLegend
AF700	CD45.1 (Ly5.1)	A20	0.5	1:400	110724	BioLegend
LIVE/DEAD™ Fixable Near‐IR Dead Cell Stain Kit	N/A	N/A	N/A	1:500*^a^*	L34976	Thermo Fisher Scientific

^*a*^ This dilution is after reconstitution with 50 µl DMSO.

## Basic Protocol 5 ADOPTIVE CD8 T CELL TRANSFER AND LCMV INFECTION

To investigate CD8 T cell differentiation during chronic LCMV infection, we provide a protocol for adoptive transfer of isolated naïve P14 cells (Basic Protocol 4 and Alternate Protocol 2) into recipient mice prior to high‐dose infection with LCMV strain CL13. Using this protocol, mice will develop a chronic LCMV infection, and the transferred LCMV‐specific CD8 T cells will expand and differentiate toward exhaustion.

**Time Considerations**

Time considerations vary significantly depending on the experience of the researcher in animal handling. Plan for 15 to 30 min per cage of five animals.

### Materials

Isolated naïve T cells for adoptive transfer (see Basic Protocol 4 or Alternate Protocol 2)
PBS, sterile (BioConcept, cat. no. 3‐05F29‐I)
Recipient mice (e.g., from B57BL/6 or P14 mouse line)
LCMV stocks (see Basic Protocol 1)

2‐ml microtubes (Sarstedt, cat. no. 72.695.500)
Recipient animal cages
Heat source of choice (e.g., warming lamp, water bath, or thermal cage)
30G syringes with long (12‐mm) needles (B.Braun, cat. no. 9151125S)
Rodent restrainer (WPI, cat. no. II‐84, or equivalent)
70% (v/v) ethanol pads

Additional reagents and equipment for animal husbandry (see Basic Protocol 6)

*NOTE*: Use recipient mice between 10 and 20 weeks of age, as they can best cope with the transient but substantial burden of a chronic LCMV infection. Use TCR‐non‐transgenic recipient mice, which match the genetic background of your donor P14 CD8 T cells, to avoid rejection if you opt for adoptive T cell transfer. Do not use female recipient mice if cells for adoptive T cell transfer are from a male donor. Do not infect P14 transgenic mice, as this may result in extensive immunopathology and viral escape through mutation of the peptide sequence recognized by the P14 TCR. The endogenous TCR repertoire from the recipient mouse is essential to prevent virus escape.

*NOTE*: Work in sterile conditions and according to local regulations of animal welfare authorities and husbandry officials.

1 Prepare the isolated naïve T cells for adoptive transfer in sterile PBS in 2‐ml microtubes.
  Adhere to the guidelines from the local veterinary office. Do not transfer more than 10,000 P14 CD8 T cells per mouse unless this is required for the specific scientific question. Exceeding this number will lead to potentially lethal immunopathology occurring from day 6 post‐infection. Injecting 200 µl P14 CD8 T cell solution per mouse is a practical volume. This results in a transfer concentration of up to 50,000 cells/ml. Depending on the research question, fewer than 10,000 cells can be transferred. The absolute numbers of cells present in the organs on the day of harvest correlate with the input at the beginning of the experiment.
2 Prepare recipient animal cages.
  It is recommended to bring all cages to the area where you will do the procedure at once to be more time efficient. This step also entails the removal of cage enrichment so the warming of the animals is more efficient afterward.
3 Warm up the recipient mice using heat source of choice prior to injections.
  This step is critical to ensure a fast and successful intravenous injection. Several options (e.g., infrared warming lamp, water bath, or thermal cage) can be used to warm the mice or only the tail of the mouse in the case of using a water bath.
4 Aspirate the appropriate volume of donor cells in PBS (see step 1) in a 30G syringe with a long (12‐mm) needle.
  Using syringes with long (12‐mm) needles simplifies the process compared to employing the 8‐mm needles normally used for intraperitoneal (i.p.) injections.
5 Restrain the mouse using a rodent restrainer according to local animal welfare regulations.
6 Disinfect the mouse tail with 70% ethanol pads and inject the donor cell solution into the tail vein of the mouse.
  The donor cell solution must be delivered into the bloodstream and not the surrounding tissue. Any form of resistance indicates mistargeting and requires immediately halting the injection.
7 Remove the syringe and stop the bleeding by gently pressing a tissue on the puncture site.
8 Transfer the mouse back to the cage.
  Move mice according to your local animal welfare regulations. You can use tunnels or cup handling for gentle and stress‐free transfers.
9 Let the mice rest for 1 day.
  Infecting mice 1 day after adoptive cell transfer is considered best practice, as the transferred cells have enough time to home to secondary lymphoid organs, where they naturally reside. The transferred cells will survive in the absence of infection, allowing longer waiting times before infection.
10 Remove LCMV stocks stored at –80°C and keep the vials on ice.
  It is critical to inject ≥2 × 10^6^ ffu LCMV CL13 per mouse to ensure a chronic infection and induction of CD8 T cell exhaustion. If you inject 200 µl, you will need an LCMV CL13 stock of ≥1 × 10^7^ ffu/ml. An intermediate infection with substantial viral titers but highly functional CD8 T cells can induce lethal immunopathology in mice or early clearance of the infection without induction of exhausted CD8 T cell lineages.
11 Repeat steps 3 to 8, replacing the donor cell solution with the desired LCMV stock to infect the recipient mice with ≥2 × 10^6^ ffu LCMV CL13. If body weight monitoring is required (see Basic Protocol 6), measure the initial body weight of the mice on the day of infection.
  Only take the virus stocks from the ice and thaw them when the animals are being warmed up for injection preparation (see step 3). Minimize the time between thawing and injection of the virus.
12 Add additional nesting material and easily accessible food (see Basic Protocol 6).

## Basic Protocol 6 ANIMAL HUSBANDRY

To assess the well‐being of mice infected with high‐dose LCMV CL13 (Basic Protocol 5), it is important to adhere to local animal welfare regulations. Here, we describe different metrics and measures to improve the well‐being of mice subjected to chronic LCMV infection. The duration of a chronic LCMV infection experiment varies depending on the scientific question. To investigate CD8 T cell exhaustion as depicted in Figure 2, 21 days post‐infection (dpi) is a good experimental end point.

**Time Considerations**

Time considerations vary significantly depending on the experience of the researcher in animal handling. Plan for 10 to 20 min per cage of five animals. It makes sense to monitor the animals once per day until the animals start to regain weight.

### Materials

Infected mice (see Basic Protocol 5)
Rodent food and water
Vaseline or other lubricant

Nesting material (e.g., tissues, straw, or wood wool)
Cage enrichment (e.g., tunnel, balcony, or mouse house)
Scale suitable for rodents (from Braintree Scientific or equivalent)
Rodent thermometer (Bioseb Lab Instruments, BIO‐TK8851 or BIO‐BRET3)

*NOTE*: Work in sterile conditions and according to local regulations of animal welfare authorities and husbandry officials.

1 Provide enough nesting material, cage enrichment, and rodent food and water for the infected mice.
  The mice will transiently fall severely sick, which manifests in restricted movement and a drop in body temperature and body weight between 6 dpi and 8 to 12 dpi. To comfort the animals, they should have enough material to build a warming nest before they fall sick and have food in reach.
2 Measure the body weight of the infected mice if required, using a scale suitable for rodents.
  Start measuring the body weight on the day of the infection. It can be expected that the body weight will start to drop only from 6 dpi onward, when the body temperature will drop as well. Scientifically, it makes no sense to measure the body weight before 6 dpi. The body weight will drop approximately until days 8 to 12 dpi. Mice chronically infected with LCMV without P14 CD8 T cell transfer will only show a slight reduction in activity and a reduction in body weight of 5% to 10%. For mice that received 10,000 P14 CD8 T cells by adoptive transfer, the maximum recommended in Basic Protocol 5, weight loss of 10% to 30% is expected.
3 Measure body temperature if required, using a rodent thermometer and Vaseline or other lubricant.
  As for the body weight measurement in step 2, it makes sense to start monitoring body temperature from 6 dpi onward. Rectal measurement is a very reliable and simple way to achieve body temperature monitoring. Use Vaseline or another lubricant to avoid unnecessary stress and pain for the animal. A severe drop from the normal temperature of 36.5° to 37.5°C to 32° to 33°C within 1 day is not unusual. Again, the drop is much more significant if the mice received P14 CD8 T cells by adoptive transfer. Most animals will recover within 2 to 3 days, which will also manifest in regained activity. Animals that do not regain temperature and that continue to lose weight are probably experiencing an intermediate infection. LCMV‐specific cells will not fully engage in exhaustion differentiation and therefore cause potentially lethal immunopathology. It is thus crucial to set termination criteria for the experiments.
4 Measure activity and pain if required.
  Activity will be reduced from 6 dpi onward and correlates very well with the body temperature. If the body temperature stays in a physiological range, signs of pain are not expected. It is still crucial to plan on potential analgesic procedures for the experiments. Keep in mind that many analgesics are immunomodulatory drugs that introduce a potentially unwanted bias in the experiments.

## Basic Protocol 7 PREPARATION OF SINGLE‐CELL SUSPENSION FROM SECONDARY LYMPHOID ORGANS

To analyze CD8 T cells and other leukocytes from secondary lymphoid organs (spleen and lymph nodes; this protocol) and in peripheral tissues (Alternate Protocol 3), the organs are harvested and processed. Then, cells can be stained for cell surface antigens and intracellular proteins and analyzed via flow cytometry (Basic Protocols 8 to 10).

**Time Considerations**

The duration of the organ harvest depends on the number of animals and the number of organs that need to be extracted per animal. Preparation of a single‐cell suspension from secondary lymphoid organs takes ∼10 min for lymph nodes and 20 min for spleens. Each additional organ will add 2 to 3 min to the total processing time.

### Materials

Infected mice (see Basic Protocol 5)
FACS buffer (see recipe)
ACK lysis buffer (see recipe)

2‐ml microtubes (Sarstedt, cat. no. 72.695.500)
70‐µm cell strainer (SmartStrainer, Miltenyi Biotec, cat. no. 130‐098‐462)
50‐ml centrifuge tubes (TPP, cat. no. 91050)
Standard tabletop centrifuge (Eppendorf AG, model no. 5910 Ri, or equivalent)

Additional reagents and equipment for mouse euthanasia and organ harvest

1 Euthanize infected mice and harvest spleen, lymph nodes, and other organs of interest into 2‐ml microtubes with 1 ml FACS buffer.
  To investigate CD8 T cell exhaustion in chronic LCMV infection, 21 dpi is a good experimental end point. Before that, the typical exhaustion phenotypes are still being established.
2 Dissociate organs by passing through a 70‐µm cell strainer placed on a 50‐ml centrifuge tube.
  You can use the flat part of a syringe (e.g., Terumo, cat. no. MDSS01SE) plunger to pass the organ through the strainer.
3 Wash the cell strainer with 10 ml FACS buffer.
4 Centrifuge the cells for 5 min at 550 × *g* and discard the supernatant.
5 Resuspend cells in 1 mL ACK lysis buffer and incubate for 5 min at room temperature to lyse erythrocytes.
6 Add 9 ml FACS buffer to the cells and centrifuge 5 min at 550 × *g*.
7 Resuspend the cells in the desired volume of FACS buffer and proceed with the antibody staining for flow cytometry (see Basic Protocol 8).
  FACS tubes (5‐ml polystyrene round‐bottom tubes, Corning, cat. no. 352054) or 96‐well round‐bottom plates (TPP, cat. no. 92097) are recommended carrier formats to distribute your cells to start the staining procedure.

## Alternate Protocol 3 PREPARATION OF SINGLE‐CELL SUSPENSION FROM PERIPHERAL TISSUES

For the isolation of CD8 T cells and other lymphocytes from peripheral tissues such as lungs, liver, or kidney, enzymatic digestion is required. This is generally not necessary for the spleen and lymph nodes (Basic Protocol 7). Following the digestion, lymphocytes are enriched by performing a 30% Percoll gradient centrifugation. The composition of the digestion cocktail must be optimized individually for every tissue. An exemplary protocol for isolating lymphocytes from lung tissue is provided.

**Time Considerations**

The duration of the organ harvest depends on the number of animals and the number of peripheral organs per animal that need to be extracted in the experiment. Preparation of a single‐cell suspension from peripheral organs is organ specific. If a digestion step and density centrifugation need to be done, the duration of Alternate Protocol 3 is extended by 2 to 4 hr compared to Basic Protocol 7.

### Additional Materials (also see Basic Protocol 7)

PBS, sterile (BioConcept, cat. no. 3‐05F29‐I), 4°C
Digestion cocktail (see recipe; make fresh)
cRPMI (see recipe)
30% (v/v) Percoll (Sigma‐Aldrich Chemie, cat. no. P1644‐1L) in PBS (BioConcept, cat. no. 3‐05F29‐I)

Perfusion syringe (BD, cat. no. 300613)
Perfusion needle (B.Braun, cat. no. 4657624B)
gentleMACS™ C tubes (Miltenyi Biotec, cat. no. 130‐093‐237)
gentleMACS™ dissociator (Miltenyi Biotec, cat. no. 130‐093‐235)
Standard tabletop centrifuge (Eppendorf AG, model no. 5910 Ri, or equivalent), room temperature and 4°C
15‐ml centrifuge tubes (TPP, cat. no. 91015)
Vacuum aspiration system (Integra or equivalent)

1 Euthanize infected mice and subsequently perform perfusion by intracardiac injection of 5 ml ice‐cold sterile PBS with a perfusion syringe and needle.
2 Harvest lungs and transfer them into gentleMACS^TM^ C tubes containing 1 ml digestion cocktail.
3 Dissociate lungs using a gentleMACS^TM^ dissociator, selecting the program m_lungs_01_02.
4 Digest the lungs for 45 min at 37°C.
5 Homogenize lungs using the gentleMACS^TM^ dissociator, selecting the program m_lungs_02_01.
6 Filter the cell suspension through a 70‐µm cell strainer placed on a 50‐ml centrifuge tube.
  You can use the flat part of a syringe (e.g., Terumo, cat. no. MDSS01SE) plunger to pass the organ through the strainer.
7 Centrifuge the cells for 5 min at 550 × *g*, discard the supernatant, and resuspend in 8 ml cRPMI.
8 Layer the cell suspension over 4 ml of 30% Percoll in PBS in a 15‐ml centrifuge tube.
9 Centrifuge the cells for 30 min at 755 × *g*, 4°C. Disable the brake of the centrifuge for this step.
10 Remove the supernatant by aspiration.
  In this step, the supernatant should not be just poured off, as de novo mixing of the Percoll layer with the lymphocytes can be toxic to the cells and will reduce the yield of the isolation.
11 Resuspend cells in 1 ml ACK lysis buffer and incubate for 5 min at room temperature to lyse erythrocytes.
12 Add 9 ml FACS buffer to the cells and centrifuge 5 min at 550 × *g*.
13 Resuspend the cells in the desired volume of FACS buffer and proceed with the antibody staining for flow cytometry (see Basic Protocol 8).

## FLOW CYTOMETRIC ANALYSIS

Multiparameter flow cytometry enables the analysis of CD8 T cells and other immune cell populations across tissues and time points during LCMV infection (Basic Protocol 5) by detecting phenotypic and functional markers with fluorochrome‑conjugated antibodies. This protocol describes a flow cytometry workflow for the detailed characterization of exhausted CD8 T cells. Several panels are provided, which cover many experimental aspects of T cell exhaustion research and are tailored to different flow cytometry analyzers or sorters.

### Time Considerations

Once the single‐cell suspensions are ready (Basic Protocol 7 and Alternate Protocol 3), allocate 1 hr for surface staining with the appropriate washing steps (Basic Protocol 8). If fixation and intracellular staining are required (Basic Protocol 8), schedule another 2 hr. If an extensive functional assessment of exhausted CD8 T cells is intended (Basic Protocol 9), schedule a total assay time of 8 hr. The initial 5‐hr incubation with the restimulating reagents is followed by both the surface and the intracellular staining procedures.

If sorting is part of the experiment (Basic Protocol 10), coordinate the preparation of the machine with your facility or allocate 1 hr to start and calibrate the sorter. Once the sorter is running, the stream has stabilized and the drop delay has been calculated, allocate 30 min for acquisition of the single‐stain controls (SSCs). Then, expect 20 min per enriched CD8 T cell fraction of a spleen if an 85‐µm nozzle is used or 10 min if a 70‐µm nozzle is used.

### Panel Design

Here, some aspects of panel design are discussed, and three exemplary panels that have successfully been used to characterize exhausted CD8 T cells are shown. Panel A is designed for a conventional analyzer or sorter (e.g., BD LSRFortessa or BD FACSAria) and covers the most important markers for definition of the different exhausted subsets in an established chronic LCMV infection (21 dpi; Table 5). Panel B is designed for spectral flow cytometry (e.g., Cytek Aurora, BD Symphony, or equivalent) and allows basic phenotyping of virus‐specific CD8 T cells but also takes other cell types into consideration (Table 6). Panel C can be used if a functional readout for a restimulation assay is needed (Basic Protocol 9 and Table 7). Given that in‐depth phenotyping of antigen‐specific CD8 T cells requires staining of many target antigens on the same cell type, some general concepts should be considered:

• Use dim fluorochromes for lineage markers (e.g., CD45, CD3, CD8).
• Choose fluorochromes with minor stain index reduction for target antigens on the same cell. Spectral similarity of two fluorochromes can lead to impaired sensitivity for one or both parameters. Therefore, it makes sense to avoid spectrally similar fluorochromes on the same cell.
• Choose fluorochromes with expected stain index reduction for target antigens expressed on mutually exclusive cell subsets.
• Use bright fluorochromes for low‐expression or unknown markers.
• Antibody titration using the cell type of interest ameliorates signal resolution and lowers the costs of the experiment.
• Panel testing is still required.

**Table 5:** Panel A: Flow Cytometry Panel for Sorting and Analysis of Exhausted CD8 T Cells from Chronic LCMV Infection Following Adoptive Cell Transfer of Congenically Marked P14 CD8 T Cells

Fluorochrome/dye*^a^*	Antigen	Clone	Concentration (mg/ml)	Working dilution	Catalog number	Vendor
BUV395	CD8a	53‐6.7	0.2	1:400	563786	BD Biosciences
BUV737	CD45.2	104	0.2	1:200	612778	BD Biosciences
Pacific Blue	CD45.1	A20	0.5	1:400	110722	BioLegend
PerCP‐Cy5.5	CX3CR1	SA011F11	0.2	1:200	149010	BioLegend
PE‐Dazzle594	CXCR6	SA051D1	0.2	1:200	151116	BioLegend
PE‐Cy7	PD‐1	29F.1A12	0.2	1:400	135215	BioLegend
APC	Ly108	330‐AJ	0.2	1:200	134609	BioLegend
LIVE/DEAD™ Fixable Near‐IR Dead Cell Stain Kit	N/A	N/A	N/A	1:500*^b^*	L34976	Thermo Fisher Scientific

^*a*^ This panel is primarily designed for sorting of P14 CD8 T cells and the different exhausted subsets present during a chronic infection and works on standard conventional sorters (e.g., BD FACSAria II) and analyzers.

^*b*^ This dilution is after reconstitution with 50 µl DMSO.

**Table 6:** Panel B: Flow Cytometry Panel for In‐Depth Analysis of Exhausted CD8 T Cells and Other Immune Cell Subsets from Chronic LCMV Infection Following Adoptive Cell Transfer of Congenically Marked P14 CD8 T Cells

Fluorochrome/dye*^a^*	Antigen	Clone	Concentration (mg/ml)	Working dilution	Catalog number	Vendor
Spark Plus UV395	XCR1 (CCXCR1)	ZET	0.2	1:200	148239	BioLegend
LIVE/DEAD™ Fixable BLUE Dead Cell Stain Kit	N/A	N/A	N/A	1:500*^b^*	L23105	Thermo Fisher Scientific
BUV496	CD4	GK1.5	0.2	1:1600	612952	BD Biosciences
BUV563	CD49b (pan‐NK cells)	HMa2	0.2	1:800	741280	BD Biosciences
BUV615	CD11c	N418	0.2	1:800	751222	BD Biosciences
BUV661	IgM	II/41	0.2	1:400	750660	BD Biosciences
BUV737	CD19	1D3	0.2	1:1600	612781	BD Biosciences
BUV805	Ly‐6C	HK1.4.rMAb	0.2	1:800	755202	BD Biosciences
BV480	CX3CR1	Z8‐50	0.2	1:400	567824	BD Biosciences
BV510	IgD	11‐26c.2a	0.2	1:400	405723	BioLegend
BV570	CD44	IM7	0.1 (lot specific)	1:400	103037	BioLegend
BV605	CD62L (L‐Selectin)	MEL‐14	0.2	1:3200	104438	BioLegend
BV650	I‐A/I‐E (MHC class II)	M5/114.15.2	0.2	1:1600	107641	BioLegend
BV711	CD186 (CXCR6)	SA051D1	0.2	1:400	151111	BioLegend
BV750	CD138 (Syndecan‐1)	281‐2	0.5	1:400	747070	BD Biosciences
RB545	CD45 (LCA)	30‐F11	0.2	1:1600	569257	BD Biosciences
Spark Blue574	CD8a	53‐6.7	0.5	1:1600	100794	BioLegend
RB613	CD95 (Fas)	Jo2	0.2	1:800	758230	BD Biosciences
RB670	CD185 (CXCR5)	2G8	0.2	1:200	771366	BD Biosciences
RB705	TCR Vα2	B20.1	0.2	1:3200	756886	BD Biosciences
RB744	CD45.1 (Ly5.1)	A20	0.2	1:400	758117	BD Biosciences
RB780	CD172a (SIRPa)	P84	0.2	1:400	755521	BD Biosciences
PE	TCF1 (TCF7)	C63D9	0.075	1:400	14456S	Cell Signaling Technology
PE‐Fire640	CD161 (NK1.1)	PK136	0.2	1:800	108770	BioLegend
PE‐Cy5	F4/80 (Ly71)	BM8	0.2	1:200	123111	BioLegend
PE‐Fire700	CD274 (PD‐L1)	10F.9G2	0.2	1:1600	124350	BioLegend
PE‐Fire810	CD279 (PD‐1)	29F.1A12	0.2	1:1600	135253	BioLegend
APC	CD11b (Mac‐1)	M1/70	0.2	1:1600	101212	BioLegend
Spark NIR685	CD3	17A2	0.5	1:800	100262	BioLegend
R718	FoxP3	3G3	0.2	1:400	567465	BD Biosciences
APC‐Cy7	CD45R/B220	RA3‐682	0.2	1:800	103224	BioLegend
APC‐Fire810	Ly‐6G	1A8	0.2	1:200	127669	BioLegend

^*a*^ This panel allows for simultaneous investigation of all major immune cell types and requires a spectral analyzer.

^*b*^ This dilution is after reconstitution with 50 µl DMSO.

**Table 7:** Panel C: Flow Cytometry Panel for Analysis of Effector Function Potential of Exhausted CD8 T Cells After Restimulation Following Adoptive Cell Transfer of Congenically Marked P14 CD8 T Cells

Fluorochrome/dye*^a^*	Antigen	Clone	Concentration (mg/ml)	Working dilution	Catalog number	Vendor
BUV395	CD62L (L‐Selectin)	MEL‐14	0.2	1:1600	569400	BD Biosciences
LIVE/DEAD™ Fixable BLUE Dead Cell Stain Kit	N/A	N/A	N/A	1:500*^b^*	L34976	Thermo Fisher Scientific
Pacific Blue	CD352 (SLAMF6, Ly108)	330‐AJ	0.5	1:200	134608	BioLegend
BV480	CX3CR1	Z8‐50	0.2	1:400	567824	BD Biosciences
BV510	CD44	IM7	0.2	1:400	103044	BioLegend
BV605	PD‐1	29F.1A12	0.2	1:800	135219	BioLegend
BV650	TNFα	MP6‐XT22	0.2	1:200	506333	BioLegend
BV711	CD186 (CXCR6)	SA051D1	0.2	1:400	151111	BioLegend
FITC	CD107a (LAMP‐1)	1D4B	0.5	1:400	121606	BioLegend
PE	TCF1 (TCF7)	C63D9	0.075	1:400	14456S	Cell Signaling Technology
PE‐Dazzle594	CD8a	53‐6.7	0.2	1:800	100762	BioLegend
PE‐Cy7	IFN‐γ	XMG1.2	0.2	1:200	557649	BioLegend
APC	IL‐2	JES6‐5H4	0.2	1:200	503810	BioLegend
AF700	CD45.1 (Ly5.1)	A20	0.5	1:400	110724	BioLegend

^*a*^ This panel works for high‐parameter conventional cytometers (e.g., BD LSRFortessa) or spectral analyzers.

^*b*^ This dilution is after reconstitution with 50 µl DMSO.

### Single‐Stain Controls

Adequate compensation or unmixing is crucial for high‐quality flow cytometry data. SSCs are therefore vital and need to be prepared with care. Although CD8 T cells are not highly autofluorescent or challenging to stain, SSCs should pe prepared upon careful consideration. Generally, SSCs should be as close to the fully stained samples as possible. Therefore, concepts to be considered include the following:

• SSCs on cells are generally better than using antibody capture beads if the target of interest is expressed at a considerable frequency.
• SSCs on beads must be chosen if the sample contains fluorescent reporters, if the entire sample needs to be available for analysis, or if the antigen of interest is expressed at very low or unknown levels or on very few cells.
• SSCs on cells or beads should be handled the same as the sample. Best practice is to prepare them in parallel with the fully stained sample.
• Reusing SSCs from an older experiment or a library is not recommended but can work especially for smaller panels or easily unmixable fluorochrome combinations. In the case of configuration changes of the machine, SSCs should not be reused.
• Tandem dyes (e.g., PE‐Cy7, BUV737) differ slightly from batch to batch and have shorter half‐life times, and special care should be taken to ensure that the SSC exactly matches the sample.

## Basic Protocol 8 Extracellular and Intracellular Staining

A step‐by‐step antibody staining procedure is provided. If the goal is to stain for intranuclear targets like transcription factors (e.g., TCF‐1, Blimp‐1, TOX), the eBioscience^TM^ FoxP3 / Transcription Factor fixation should be used. If only cytoplasmic or endosomal proteins are targeted, a milder fixation (e.g., BD Cytofix/Cytoperm) is preferred. If reporter systems are involved (e.g., eGFP), it is important to know that permeabilization and fixation will lead to total loss or significant reduction of the GFP signal because the reagents will damage the reporter proteins or the fluorescent protein will exit the cell. Anti‐GFP antibodies will not help in this case. The signal might be retained if the reporter fluorochrome is anchored to a cellular compartment or if it has an intranuclear localization domain (e.g., Cre recombinase). Otherwise, fixation prior to permeabilization can help to prevent the loss of the protein. Nevertheless, combining intracellular or intranuclear staining with retention of a cytosolic reporter fluorochrome is very challenging and will lead to suboptimal resolution of either of the signals in most cases. The basic fixation and intranuclear staining protocol is depicted below.

### Materials

Samples of interest (see Basic Protocol 7 or Alternate Protocol 3)
FACS buffer (see recipe), room temperature and 4°C
Viability dye of choice (see panels in Tables 5 to 7, or equivalent)
PBS (BioConcept, cat. no. 3‐05F29‐I), 4°C
Fluorescently labeled antibodies of choice (see panels A, B, and C in Tables 5, 6, and 7, respectively, or equivalent)
Fluorescently labeled pMHC‐I tetramers (see Basic Protocol 3; optional)
eBioscience^TM^ FoxP3 / Transcription Factor Staining Buffer Set (Thermo Fisher Scientific, cat. no. 00‐5523‐00), including Fixation / Permeabilization Concentrate, Fixation / Permeabilization Diluent, and Permeabilization Buffer (10×)
ddH_2_O
FACS tubes (5‐ml polystyrene round‐bottom tubes, Corning, cat. no. 352054) and/or 96‐well round‐bottom plates (U or V bottom; TPP, cat. no. 92097, or equivalent)

*NOTE*: Unless mentioned otherwise, “washing” means topping up the current incubation volume with FACS buffer, centrifuging the samples for 5 min at 550 × *g* (standard tabletop centrifuge, Eppendorf AG, model no. 5910 Ri, or equivalent) and discarding the supernatant.

*NOTE*: Until the cells are fixed, all procedures should be done at 4°C or on ice unless stated otherwise.

*NOTE*: Acquisition and analysis of the samples are outside the scope of this article. It is highly recommended to discuss the choice of instrument with the staff of your facility before planning and running the experiment.

#### Extracellular staining

1 Transfer the samples of interest into FACS tubes or wells of 96‐well round‐bottom plates (if the analyzer or sorter of choice provides a plate‐reading option).
2 Wash the cells with cold FACS buffer.
3 Dilute the viability dye of choice in cold PBS, add 50 µl per sample, and resuspend the cell pellet by pipetting.
  If Fc receptor blockade (with Fc receptor–blocking antibody, TrueStain FcX PLUS (anti‐mouse CD16/CD32), BioLegend, cat. no. 156604) or similar blocking steps are desired, perform these together with the viability staining and before the antibody staining.
4 Incubate the samples for 10 min at 4°C.
  If the duration between harvest and analysis needs to be minimized, the viability staining can be done together with the rest of the staining in FACS buffer. However, many viability dyes bind amine groups present on proteins. Therefore, the viability dye should be added to the antibody staining mix (see step 5) only seconds before applying it to the cells. Otherwise, the dye will react with the FBS in the FACS buffer, which limits the availability of the dye for the cells.
5 Prepare the antibody staining mix with fluorescently labeled antibodies of choice (see panels A to C in Tables 5 to 7) with the respective dilutions in cold FACS buffer. If staining for endogenous pMHC‐I‐specific CD8 T cells will be performed, add the respective fluorescently labeled pMHC‐I tetramer into the mix at the respective dilution.
6 Wash the cells.
  The washing steps can be repeated to obtain cleaner results at the expense of cell numbers and time.
7 Remove the residual volume by placing the tubes/plates upside down on tissue paper.
  This avoids unnecessary dilution of the antibody mix in step 8 and ensures comparable antibody concentrations between samples. This is especially important if comparison of marker expression between samples is of interest.
8 Add 50 µl of the prepared antibody staining mix (see step 5) per sample and resuspend the cell pellet.
9 Incubate the cells for 30 min at 4°C in the dark.
10 Wash the cells.
  The samples are ready to be analyzed or, in the case of intracellular targets, ready to be fixed and permeabilized (see steps 11 to 21).

#### Fixation and intracellular staining

11 Wash the cells.
12 Mix the Fixation / Permeabilization Concentrate and the Fixation / Permeabilization Diluent from the eBioscience™ Foxp3 / Transcription Factor Staining Buffer Set together at a ratio of 1:4.
  If intracellular but not intranuclear staining is desired, use the BD Cytofix/Cytoperm kit (BD Biosciences, cat. no. 554714) instead of the eBioscience™ Foxp3 / Transcription Factor Staining Buffer Set.
13 Add 300 µl of the mixed fixation buffer per FACS tube or 50 µl per well and resuspend the cells.
14 Incubate for 30 min at 4°C in the dark.
15 Prepare permeabilization buffer by diluting the Permeabilization Buffer (10×) from the eBioscience™ Foxp3 / Transcription Factor Staining Buffer Set at a 1:10 ratio in ddH_2_O.
16 Prepare the antibody staining mix for the intracellular targets with fluorescently labeled antibodies of choice (see panels A to C in Tables 5 to 7) at the respective dilutions in the diluted Permeabilization Buffer.
17 Wash the cells in Permeabilization Buffer.
  After fixation, the cell pellets might not be visible anymore by eye. Fixatives can make the cells transparent due to changes in the refractive index of cell membranes. Additionally, the cells get more compact due to fixation, making the overall size of the pellet smaller.
18 Remove the residual volume by placing the tubes/plates upside down on tissue paper.
19 Add 50 µl of the prepared antibody staining mix (see step 16) per sample and resuspend the cell pellet.
20 Incubate the samples for 60 min at room temperature in the dark.
21 Wash the cells at least once with FACS buffer.
  The washing steps can be repeated to obtain better results at the expense of cell numbers and time.
  The samples are ready to be analyzed.

#### Understanding results

Please see Figure 5.

**Figure 5:**
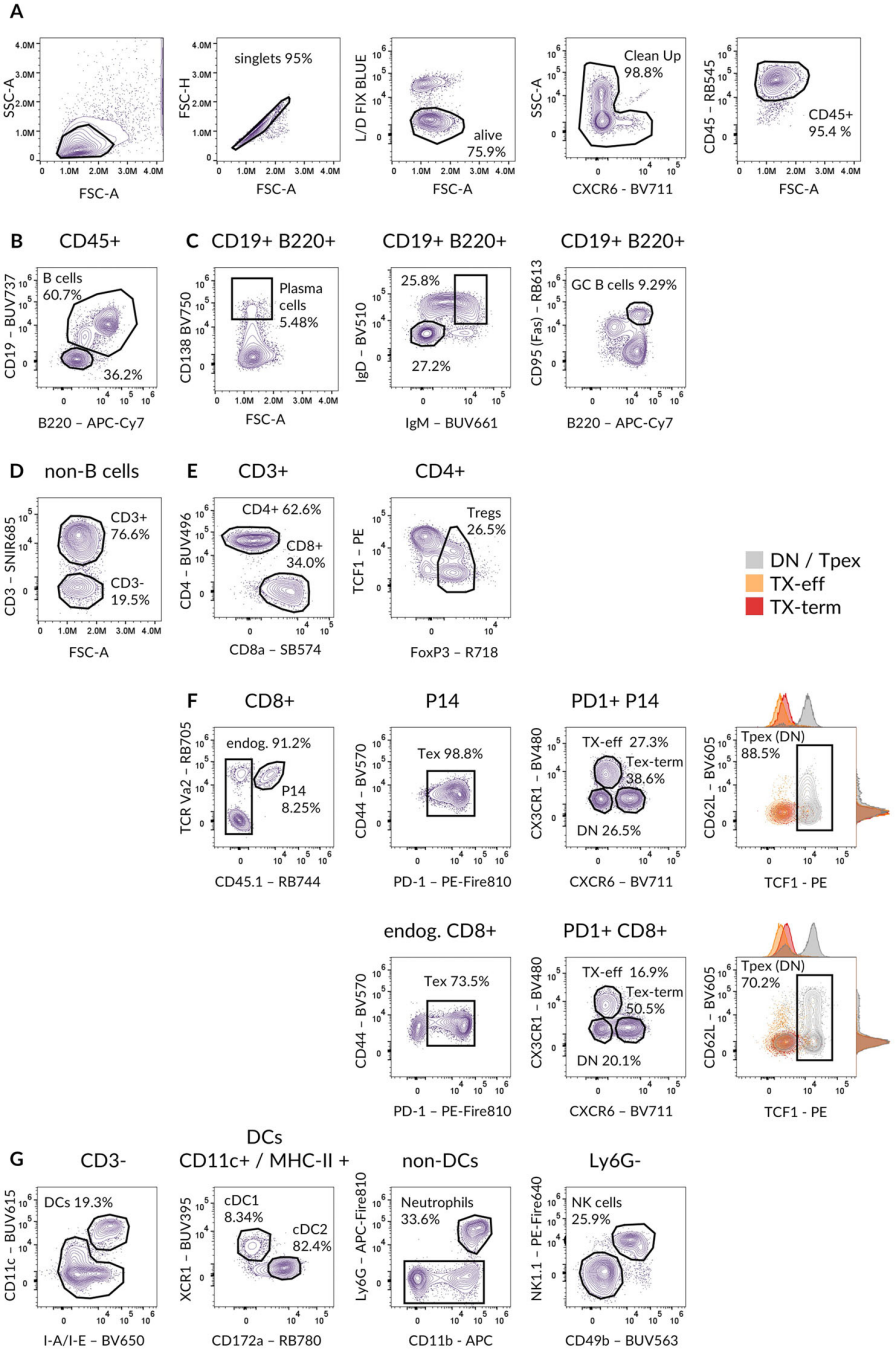
Flow cytometry gating strategy for exhausted CD8 T cells for spectral cytometry or sorting. This gating strategy is based on panel B (see Table 6) and is showcased on splenocytes isolated from a C57BL/6 mouse infected with a high dose of LCMV CL13. Prior to the infection, 10,000 naïve P14 CD8 T cells were adoptively transferred. (**A**) Standard gating strategy excluding doublets and dead cells, including a “clean‐up” gating to exclude events that are artifacts, autofluorescent cells, or antibody aggregation. Gating on CD45^+^ cells builds the basis for further immunophenotyping. (**B**) Distinguishing B cells from the rest of the immune cell types by the B cell lineage markers CD19 and B220 allows for (**C**) further B cell analysis and gating on plasma cells (CD138^high^), naïve B cells (IgM^+^ / IgD^+^), isotype‐switched B cells (IgM^−^ / IgD^−^), and germinal‐center B cells (Fas^high^ / B220^high^). (**D**) From the CD19^−^ / B220^−^ fraction, cells are divided into CD3^+^ T cells and CD3^−^ cells. (**E**) T cells are further divided into CD4^+^ and CD8^+^ T cells. Treg cells are distinguished from the conventional CD4 T cells by the expression of the key transcription factor FoxP3. (**F**) CD8 T cells are split into the transferred P14 cells or endogenous CD8 T cells, and the PD‐1^+^ fractions are subsequently divided into the three main TX subsets, namely Tpex (gray), TX‐eff (orange), and TX‐term (red). (**G**) From the CD3^−^ fraction, dendritic cells (DCs) are gated by expression of the markers CD11c and MHC‐II (I‐A / I‐E). DCs are further split into cDC1 (XCR1^+^) and cDC2 (CD172a^+^). Gated on the non‐DC fraction, neutrophils are detected using Ly6G and CD11b. Lastly, double positivity for NK1.1 and CD49b identifies natural killer (NK) cells.

## Basic Protocol 9 Restimulation analysis

Phenotyping of exhausted CD8 T cells as depicted in Figure 5 does not reveal the functional capacity that these cells still have. To assess this, analysis of effector functions such as cytokine production or degranulation capacity is required. For intracellular cytokine staining, splenocytes are restimulated *ex vivo*. For restimulation of P14 CD8 T cells, different kinds of reagents can be used: (1) polyclonal activators, such as phorbol‐12‐myristate‐13‐acetate (PMA) and ionomycin or monoclonal antibodies against CD3 with or without antibodies against costimulatory molecules such as CD28, or (2) TCR‐dependent activators, such as the cognate peptide gp33‐41 (gp33). *Ex vivo* stimulation of splenocytes is performed in the presence of a protein transport inhibitor to allow the accumulation of the cytokines of interest inside the cell and, if desired, in the presence of anti‐CD107a antibody to assess the degranulation. Following stimulation, cells can be stained for cell surface antigens and intracellular proteins and cytokines and analyzed by flow cytometry. To compare cytokine production and degranulation potential in response to stimulation, a negative control of cells that are cultured and stained in parallel, but did not receive stimulation, is required.

### Materials

Samples of interest (see Basic Protocol 7 or Alternate Protocol 3)
cRPMI (see recipe)
gp33‐41 (gp33) peptide (EMC Microcollections, cat. no. BAP‐206) or eBioscience™ Cell Stimulation Cocktail (500X) (Thermo Fisher Scientific, cat. no. 00‐4970‐93)
eBioscience™ Protein Transport Inhibitor Cocktail (500X) (Thermo Fisher Scientific, cat. no. 00‐4980‐03)
Anti‐CD107a monoclonal antibody (from panel C; see Table 7; optional)
FACS buffer (see recipe)
Fluorescently labeled antibodies of choice (see panel C in Table 7 or equivalent)
BD Perm/Wash™ Buffer (10X) (BD Biosciences, cat. no. 554723)
1× BD Perm/Wash™ Buffer (10X) (BD Biosciences, cat. no. 554723) in PBS (BioConcept, cat. no. 3‐05F29‐I)

FACS tubes (5‐ml polystyrene round‐bottom tubes, Corning, cat. no. 352054) and/or 96‐well round‐bottom plates (U or V bottom; TPP, cat. no. 92097, or equivalent)
Standard tabletop centrifuge (Eppendorf AG, model no. 5910 Ri, or equivalent)
37°C, 5% CO_2_ cell culture incubator (Heracell™, Thermo Fisher Scientific, cat. no. 50116047, or equivalent)

*NOTE*: Acquisition and analysis of the samples are outside the scope of this article. It is highly recommended to discuss the choice of instrument with the staff of your facility before planning and running the experiment.

1 Transfer ∼10^6^ fresh splenocytes into a FACS tube. Transfer the same number of cells into a separate FACS tube for the negative control.
2 Centrifuge the cells for 5 min at 550 × *g*, discard the supernatant, and resuspend the cells in 1 ml cRPMI.
3 Depending on the desired type of stimulation, either add 1 µl gp33‐41 (gp33) peptide (1 µM) or 2 µl eBioscience™ Cell Stimulation Cocktail (100X; containing PMA and ionomycin) to the cells. Also add 2 µl eBioscience™ Protein Transport Inhibitor Cocktail (500X; containing brefeldin A and monensin).
4 If desired, add 2.5 µl anti‐CD107a monoclonal antibody to the stimulated and non‐stimulated cells.
5 Incubate the cells for 5 hr at 37°C, 5% CO_2_.
6 Centrifuge the cells for 5 min at 550 × *g* and wash twice in FACS buffer.
7 Add 50 µl of mix of fluorescently labeled antibodies of choice (see panel C in Table 7) in FACS buffer per sample for surface staining and resuspend the cell pellet.
8 Incubate the cells for 30 min at 4°C in the dark.
  Cell surface staining is performed with live cells prior to fixation and permeabilization, as these steps can compromise antibody binding.
9 Centrifuge the cells for 5 min at 550 × *g* and wash once in FACS buffer.
10 Resuspend the cells in 100 µl BD Perm/Wash™ Buffer (10X).
11 Incubate the cells for 20 min at 4°C in the dark.
12 Centrifuge the cells for 5 min at 550 × *g* and wash twice in 1× BD Perm/Wash™ Buffer in PBS.
13 Add 50 µl of the prepared antibody staining mix in 1× BD Perm/Wash per sample and resuspend the cell pellet.
14 Incubate the cells for 30 min at 4°C in the dark.
15 Centrifuge the cells for 5 min at 550 × *g* and wash once in FACS buffer.
16 Resuspend the cells in 200 µl FACS buffer prior to flow cytometric analysis.

#### Understanding results

Please see Figure 6.

**Figure 6:**
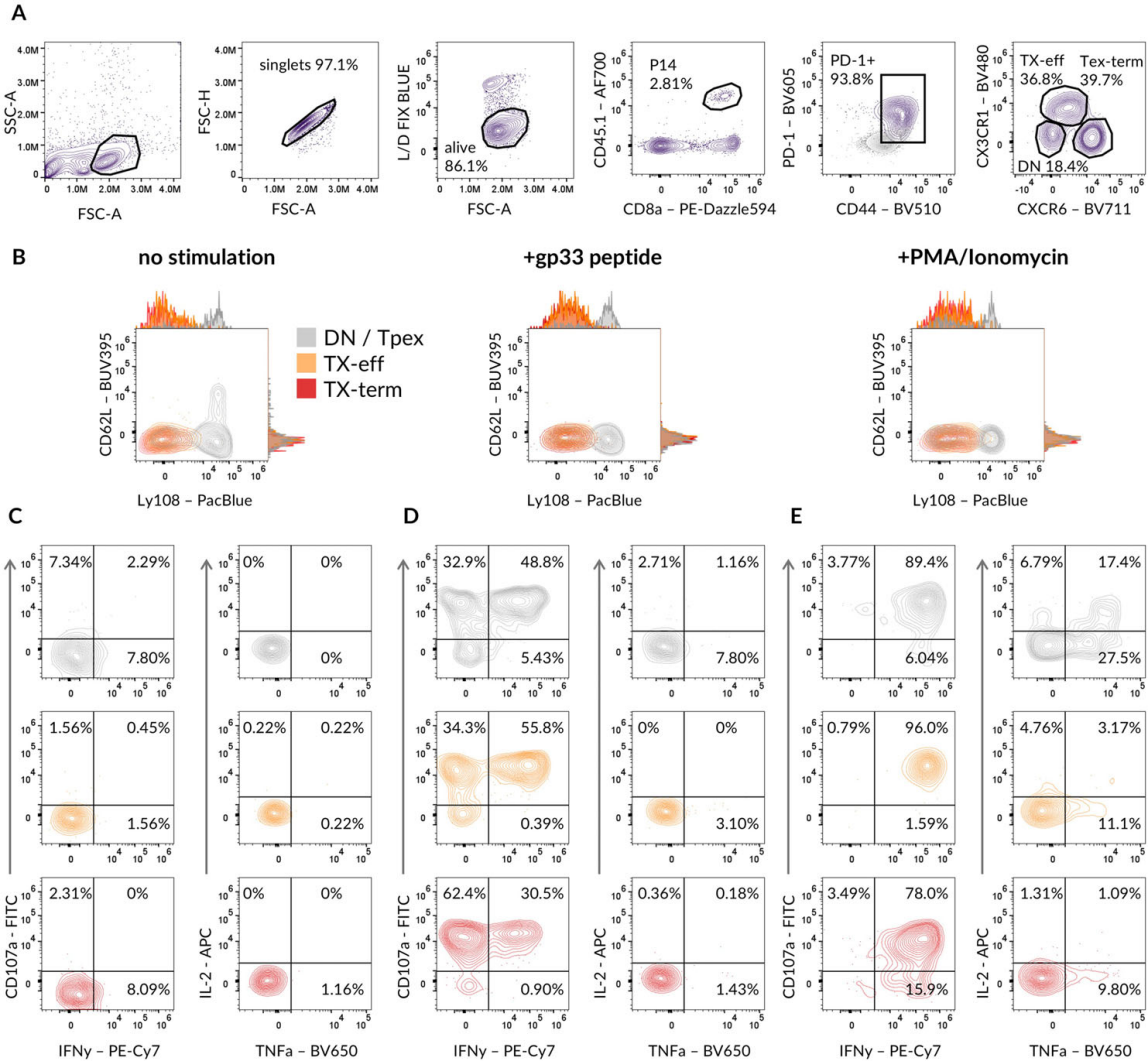
Flow cytometry gating strategy for exhausted and restimulated CD8 T cells. This gating strategy is based on panel C (see Table 7) and is showcased on splenocytes isolated from a C57BL/6 mouse infected with a high dose of LCMV CL13. Prior to the infection, 10,000 naïve P14 CD8 T cells were adoptively transferred. (**A**) Standard gating strategy excluding doublets and dead cells and focusing on the transferred CD8a^+^ T cells is shown. Comparing P14 CD8 T cells (deep purple) to naïve (CD62L^+^) endogenous cells showed CD8 T cell exhaustion based on elevated PD‐1 expression. Gating the three main exhausted subsets as depicted builds the basis for further analysis. The standard gating is shown for the gp33‐pulsed condition and is representative of all conditions. (**B**) Expression of Ly108, a proxy for the key transcription factor TCF1, confirms that the DN fraction (CX3CR1^−^ / CXCR6^−^) (gray) represents Tpex cells, whereas TX‐eff (orange) and TX‐term (red) lack expression of Ly108. The three subsets are compared in two different restimulation conditions with an unstimulated control included. (**C**) The unstimulated control shows negligible signals for both the degranulation marker CD107a and the effector cytokines IFN‐γ, IL‐2, and TNFα and therefore serves as a reference for the gating strategy. (**D**) The TCR engagement–dependent effector and degranulation potentials are assessed by stimulating CD8 T cells with their cognate antigen gp33. All TX subsets partially upregulate CD107a expression upon restimulation. Additionally, secretion of IFN‐γ is initiated upon restimulation in all subsets to different degrees. In contrast, all subsets fail to produce IL‐2 and TNFα. (**E**) Stimulation with PMA/ionomycin bypasses the TCR and thereby reflects the cell's inherent potential to perform effector functions. Here, almost all TX cells express CD107a and IFN‐γ. In comparison to the peptide restimulation condition, elevated levels of both IL‐2 and TNFα are detected with the highest frequencies in Tpex and the lowest frequencies in TX‐term.

The degranulation potential of the TX subsets is measured by the percentage of CD107a^+^ cells. The percentage of IFN‐γ^+^, TNFα^+^, or IL‐2^+^ cells indicates the effector potential of the TX subsets.

Unstimulated control samples should show no or very little signal for any of the markers and serve as reference for the gating strategy. The TCR engagement–dependent effector and degranulation potential can be assessed by stimulating the CD8 T cells with their cognate antigen gp33 when presented on MHC‐I by dendritic cells or other antigen‐presenting cells. The effect of co‐inhibitory receptor engagement influencing the activation strength via the TCR can be assessed under this activation conditions as well. In contrast, stimulation with PMA/ionomycin bypasses the TCR and thereby reflects a cell's inherent potential rather than its responsiveness to antigen engagement.

Tex cells lose their effector functions in a progressive and hierarchical order during chronic infection, with IL‐2 and TNFα lost first (Fuller & Zajac, 2003; Shin & Wherry, 2007; Wherry et al., 2003). Only Tpex still show partial expression of IL‐2 in response to stimulation with gp33 (Fig. 6D). Stimulation with PMA/ionomycin significantly increases IL‐2 expression, indicating remaining potential for IL‐2 production, which is restrained by intrinsic and extrinsic inhibition of TCR signaling (Fig. 6E). TX‐eff and TX‐term cells almost completely lose their ability to produce IL‐2. Similarly, TNFα expression is severely compromised in all Tex subsets in response to gp33 (Fig. 6D). TX cells retain inherent potential, although limited in the case of TX‐eff and TX‐term cells, to produce TNFα, as demonstrated by stimulation with PMA/ionomycin. In contrast, all Tex subsets are fully capable of expressing the effector cytokine IFN‐γ (Fig. 6E). Even in a TCR‐dependent manner, robust production of IFN‐γ is induced in all Tex subsets. However, TX‐term show significantly lower IFN‐γ expression compared to Tpex and TX‐eff, demonstrating a correlation with the severity of exhaustion. In addition, a high proportion of TX cells still retain the ability to degranulate, as measured by CD107a, irrespective of being stimulated by gp33 or PMA/ionomycin.

## Basic Protocol 10 Sorting

To analyze exhausted CD8 T cell subsets for restimulation and metabolic assays or genetic, epigenetic, and transcriptional analysis, cells can be isolated by fluorescence‐based cell sorting. The same principles apply as for other flow cytometry methods, whereby sterility, cell viability, and cell retrieval rates play an especially important role. Therefore, it is essential to always work under sterile conditions, on ice, and as swiftly as possible. It makes sense to work in teams to minimize the processing time. The panel provided here (Table 5) works on a five‐laser BD FACSAria II or any other sorter with the same or better optical configuration. A spectral sorter is not needed for sorting the main subtypes of exhausted CD8 T cells. It is highly recommended to discuss the sorter of choice with the staff of your facility.

### Materials

ACK lysis buffer (see recipe)
Fluorescently labeled antibodies of choice (see panel A in Table 5, except without viability dye)
Compensation beads (UltraComp eBeads^TM^ Spectral Unmixing Beads, Thermo Fisher Scientific, cat. no. U20250, or equivalent)
MACS buffer (see recipe)

Collection tubes
Cell sorter with optimal nozzle (BD FACSAria II or equivalent)

Additional reagents and equipment for harvesting and preparing organs (see Basic Protocol 7) and enriching cells of interest using MojoSort™ (see Alternate Protocol 2; optional)

*NOTE*: Acquisition and analysis of the samples are outside the scope of this article. It is highly recommended to discuss the choice of instrument with the staff of your facility before planning and running the experiment.

1 Harvest and prepare organs according to Basic Protocol 7.
2 Optional: Enrich cells of interest using the MojoSort™ Mouse CD8 T Cell Isolation Kit (see Alternate Protocol 2). If no enrichment is done, perform erythrocyte lysis by incubating the cell suspension for 5 min in ACK lysis buffer.
  This optional step is recommended if only CD8 T cells have to be sorted. Enriching the cells prior to sorting will substantially decrease the sorting time, which results in an overall better result for the experiment.
3 Perform extracellular staining with fluorescently labeled antibodies of choice (see panel A in Table 5) according to Basic Protocol 9, steps 1 to 10, but under sterile conditions and without the cell viability dye.
  To account for cell viability at the time of the sort, do not use a viability dye during this step, but rather add a nucleic acid stain such as Sytox^TM^ Deep Red Nucleic Acid Stain (Thermo Fisher Scientific, cat. no. S11381) to the cell suspension (1:1000) shortly before sorting. This enables the detection and exclusion of cells that died during the sorting process. Incorporating this step will increase the frequency of viable cells among the sorted populations.
  For the enriched CD8 T cell fraction of a whole spleen, 100 µl staining mix is recommended.
4 Prepare SSCs with compensation beads.
  The same panel design principles apply as discussed before (see Panel Design in the protocol introduction). However, it is worth mentioning that compensation issues need to be resolved in real time and cannot be changed retroactively. Therefore, difficult fluorophore combinations should be avoided, and the panel performance should be confirmed before the actual experiment. The provided panel A in Table 5 can be used to sort exhausted CD8 T cell subsets.
  Preparation of SSCs should be done simultaneously with preparation of the fully stained samples to guarantee comparability. Distribute the beads (UltraComp eBeads^TM^ Spectral Unmixing Beads, Thermo Fisher Scientific, cat. no. U20250, or equivalent) into wells of a 96‐well plate and add the respective antibodies per well. The beads are highly concentrated, so it is recommended to use one drop of beads for more than one SSC (up to three is recommended) for economic reasons. If you decide to dilute the beads, dilute them in FACS buffer, distribute 50 µl per well, and add the antibody. Make sure that the concentration of the antibody for the SSCs is equal to or higher than the concentration of the antibody in the fully stained samples. Incubate and wash the SSCs in exactly the same way as the fully stained samples.
5 Resuspend the cell pellet in MACS buffer.
  Compared to use with an analyzer, the cell concentration in the sample is more critical for sorting. A low concentration will prolong the acquisition time, which translates into lower viability. A very high concentration will lead to clumping of cells and cell death. From a technical perspective, a highly concentrated cell suspension will result in higher abort rates, as purity requirements can no longer be met. As a rule of thumb, resuspend the cells in 400 µl MACS buffer for the enriched CD8 T cell fraction of a whole spleen.
6 Prepare collection tubes for the sorted cells containing MACS buffer.
  Choose the specific collection tubes and collection buffer depending on the subsequent steps. Ensure that the collection tubes are cooled to minimize a drop in viability of the sorted cells.
7 Sort the cells of interest using an optimal nozzle size that minimizes shear stress while maintaining efficient flow rates.
  For P14 CD8 T cells or lymphocytes in general, an 85‐µm nozzle is recommended.
8 At the end of the sort, take an aliquot of the sorted sample (∼10 µl), dilute it in 100 to 200 µl MACS buffer, and re‐analyze it to assess the sorting purity.
  The cells are ready for subsequent use.

#### Understanding results

Please see Figure 7.

**Figure 7:**
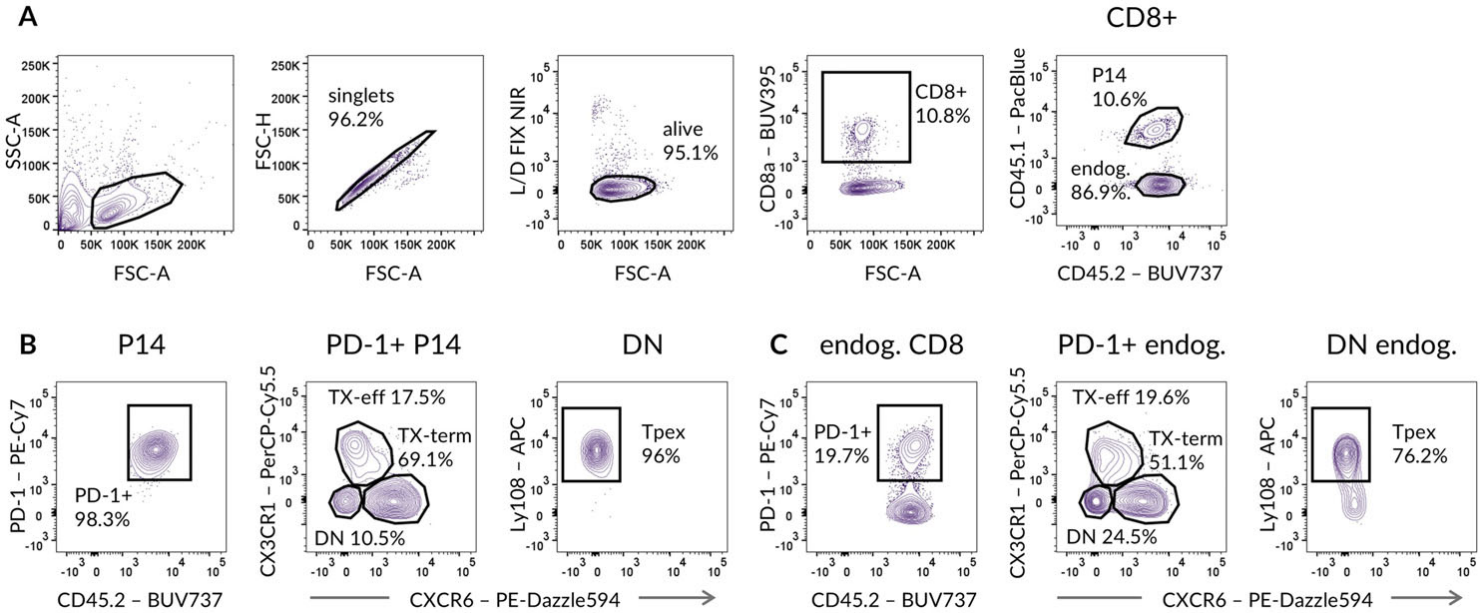
Flow cytometry gating strategy for exhausted CD8 T cells for conventional cytometry or sorting. This gating strategy is based on panel A (see Table 5) and is showcased on splenocytes isolated from a C57BL/6 mouse infected with a high dose of LCMV CL13. Prior to the infection, 10,000 naïve P14 CD8 T cells were adoptively transferred. (**A**) Standard gating strategy excluding doublets and dead cells and focusing on CD8a^+^ T cells is shown. Distinction between the transferred LCMV antigen–specific P14 CD8 T cells and endogenous CD8 T cells is based on different congenic marker expression. (**B**) High levels of PD‐1 and CD44 expression on P14 CD8 T cells indicate viral persistence and exhausted CD8 T cells. Differentiation of exhausted CD8 T cells into the three main subsets Tpex, TX‐eff, and TX‐term requires analysis of the two chemokine receptors CX3CR1 and CXCR6. The double‐negative fraction represents the Tpex cells, which are positive for Ly108 (SLAMF6, CD352), a surface antigen that correlates with the key transcription factor TCF1. Surface staining for Ly108 therefore allows the analysis and sorting of Tpex cells without the need for intracellular staining. (**C**) Phenotyping of the PD‐1^+^ fraction of the endogenous CD8 T cells reveals very similar phenotypes compared to the transferred P14 CD8 T cells.

## REAGENTS AND SOLUTIONS

### Ammonium‐chloride‐potassium (ACK) lysis buffer

150 mM ammonium chloride (NH_4_Cl; Sigma‐Aldrich Chemie, cat. no. 31107)
10 mM potassium bicarbonate (KHCO_3_; Sigma‐Aldrich Chemie, cat. no. P9144)
0.1 mM ethylenediaminetetraacetic acid (Na_2_EDTA; Applichem/Axonlab, cat. no. A1104)
ddH_2_O
Adjust pH to 7.2 to 7.4 by adding 1 M NaOH or 1 M HCl
Store ≤6 months at 4°C

### cRPMI

RPMI 1640 (Life Technologies, cat. no. 31870025)
10% (v/v) FBS (Life Technologies, cat. no. 10270106)
33 µM beta‐mercaptoethanol (Life Technologies, cat. no. 31350010)
2 mM GlutaMax (Life Technologies, cat. no. 35050‐038)
20 mM HEPES buffer (Chemie Brunschwig, cat. no. P05‐01100)
1% (v/v) non‐essential amino acids (NEAA; Life Technologies, cat. no. 11140‐035)
1% (v/v) sodium pyruvate (NaP; Life Technologies, cat. no. 11360039)
100 U/ml penicillin and 10 µg/ml streptomycin (Life Technologies, cat. no. 15070063)
Store ≤1 month at 4°C

### Digestion cocktail

600 U/ml collagenase IV (Sigma‐Aldrich Chemie, cat. no. C5138‐25MG)
200 U/ml deoxyribonuclease (DNAse) I (Huberlab, cat. no. A3778.0500)
cRPMI (see recipe)
Prepare fresh immediately before use

### FACS buffer

20 ml FBS (Life Technologies, cat. no. 10270106)
10 ml 0.5 M EDTA (Sigma‐Aldrich Chemie, cat. no. E7889‐100ML)
500 ml PBS (BioConcept, cat. no. 3‐05F29‐I)
Store ≤2 weeks at 4°C

### Infection medium

Minimal essential medium (MEM; BioConcept, cat. no. 1‐31F01‐I)
2% (v/v) FBS (Life Technologies, cat. no. 10270106)
1% (v/v) PSG (see recipe)
Store ≤1 month at 4°C

### MACS buffer

10 ml FBS (Life Technologies, cat. no. 10270106)
2 ml 0.5 M EDTA (Sigma‐Aldrich Chemie, cat. no. E7889‐100ML)
500 ml PBS (BioConcept, cat. no. 3‐05F29‐I)
Store ≤2 weeks at 4°C and keep sterile

### Maintenance medium

MEM (BioConcept, cat. no. 1‐31F01‐I)
5% (v/v) FBS (Life Technologies, cat. no. 10270106)
1% (v/v) PSG (see recipe)
Store ≤ 1 month at 4°C

### Methylcellulose, 2%

10 g methylcellulose (1500 cP; Sigma‐Aldrich Chemie, cat. no. H8384‐25G)
500 ml ddH_2_O
Stir overnight at 4°C
Autoclave
Stir again until completely dissolved
Store ≤6 months at 4°C

### Overlay solution

Mix 2% (w/v) methylcellulose (see recipe) with 2× MEM (Thermo Fisher Scientific, cat. no. 21430020) supplemented with 10% (v/v) FBS (Life Technologies, cat. no. 10270106) and 2× PSG 1:1 to get a 1% methylcellulose in 1× MEM solution. Store ≤2 weeks at 4°C.

### Penicillin‐streptomycin‐glutamine (PSG)

100 U/ml penicillin and 10 µg/ml streptomycin (Life Technologies, cat. no. 15070063)
2 mM L‐glutamine (BioConcept, cat. no. 5‐10K00‐H)
500 ml PBS (BioConcept, cat. no. 3‐05F29‐I)
Store ≤1 year at –20°C

## COMMENTARY

The detailed protocols provided in this article should form a solid basis for experimental endeavors in the context of acute and chronic LCMV infection and contribute to the success and robustness of experimental research in the field of CD8 T cell exhaustion. We hope that our work helps in harmonizing experimental procedures and supports groups that are about to embark on research in the fascinating field of chronic infections and CD8 T cell exhaustion.

### Author Contributions

**Dominique Stark**: Conceptualization; writing—original draft; writing—review and editing; visualization. **Marie‐Elen Tuchel**: Conceptualization; writing—original draft; writing—review and editing; visualization. **Gautier Stehli**: Conceptualization; writing—original draft; writing—review and editing; visualization. **Alessandro Genovese**: Conceptualization; writing—original draft; writing—review and editing. **Isaak Quast**: Conceptualization; writing—review and editing; project administration; supervision. **Annette Oxenius**: Conceptualization; writing—review and editing; funding acquisition; project administration; supervision.

### Conflict of Interest

The authors declare no conflict of interest.

## Data Availability

The datasets generated for this article to showcase the quality of the protocols are not publicly accessible but are available from the corresponding authors upon reasonable request.
